# Identification of circRNA-associated ceRNA networks using longissimus thoracis of pigs of different breeds and growth stages

**DOI:** 10.1186/s12864-022-08515-7

**Published:** 2022-04-11

**Authors:** Xiaona Zhuang, Zekun Lin, Fang Xie, Junyi Luo, Ting Chen, Qianyun Xi, Yongliang Zhang, Jiajie Sun

**Affiliations:** Guangdong Provincial Key Laboratory of Animal Nutrition Control, National Engineering Research Center for Breeding Swine Industry, Guangdong Laboratory for Lingnan Modern Agriculture, South China Agricultural University, Guangzhou, 510642 Guangdong China

**Keywords:** CircRNA, Meat quality, C_2_C_12_, circKANSL1L, RNA-seq

## Abstract

**Background:**

Long-term artificial selection for growth rate and lean meat rate has eventually led to meat quality deterioration. Muscle fiber type is a key factor that markedly affects meat quality. circRNAs have been reported to participate in diverse biological activities, including myofiber growth and development; thus, we herein compared porcine circRNA transcriptome between oxidative and glycolytic muscle tissues.

**Results:**

Longissimus thoracis muscle tissues were obtained from Lantang and Landrace pigs at birth (LT1D and LW1D, respectively) and 90 postnatal days (LT90D and LW90D, respectively). Hematoxylin and eosin staining and quantitative real-time PCR revealed that all structural traits of the muscle showed large variations between different breeds and growth stages. In total, 329 known miRNAs and 42,081 transcript candidates were identified; 6,962 differentially expressed transcripts were found to play a key role in myogenesis by gene ontology and Kyoto Encyclopedia of Genes and Genomes pathway analyses. In addition, 3,352 circRNAs were identified using five predicting algorithms, and 104 circRNA candidates were differentially expressed. Integrated analysis of differentially expressed miRNAs, mRNAs, and circRNAs led to the identification of 777, 855, and 22 convincing ceRNA interactions in LT1D vs. LT90D, LW1D vs. LW90D, and LT90D vs. LW90D, respectively. Finally, we identified a circRNA candidate circKANSL1L, which showed high homology between mice and pigs, and it was found to inhibit the proliferation of C_2_C_12_ cells but promote their differentiation.

**Conclusions:**

We identified genome-wide circRNAs in 0- and 90-day-old Lantang and Landrace pigs by RNA-seq and found that circRNAs were abundant, differentially expressed, and associated with myogenesis. Our results should serve as a reference for future studies on pork quality.

**Supplementary Information:**

The online version contains supplementary material available at 10.1186/s12864-022-08515-7.

## Background

Over the past few decades, modern pig breeding programs have primarily focused on the genetic improvement of economically important traits [1]. Consequently, commercial pig breeds with highly desirable features, such as rapid growth rate [2], favorable feeding behavior [3], higher weight gain [2], lean meat content [2], excellent fertility [4], and enhanced disease resistance [5], now exist. Certain meat quality traits, such as color, marbling, tenderness, juiciness, and flavor, play an essential role in the consumer acceptance of pork; however, long-term selection has been reported to markedly affect these traits [6]. Pork quality is a complex feature that is associated with various physical and biochemical parameters, including environmental conditions, pre-slaughter handling, slaughter procedure, energy metabolism, lipid deposition, and myofiber characteristics [7, 8]. In general, muscle fibers strongly influence meat quality, and they can be differentiated into oxidative and glycolytic types depending on contractile and metabolic properties as well as morphological traits [9].

The growth and development of myofibers involves ontogenesis during distinct embryonic stages, as well as hypertrophy and conversion in postnatal stage [10]. Such biological processes are controlled by several myogenic regulatory factors [11], signaling pathways [12], genes, and noncoding RNAs (ncRNAs) [13] via diverse mechanisms. Circular RNAs (circRNAs) are a large class of ncRNAs with covalently closed continuous loop structures, and they are produced from precursor mRNA back-splicing [14]. Recent research indicates that circRNAs play a key role in myogenesis in various organisms [15]. They are dynamically expressed and particularly abundant in muscle tissues across many species, including humans [16], monkeys [17], bovine [18], goats [19], sheep [20], pigs [21], chicken [22], and mice [23]. Although the functions of circRNAs remain largely unexplored, their most important role is to serve as miRNA sponge and promote mRNA stability or protein production [24]. In this study, we compared porcine circRNA transcriptome between oxidative and glycolytic skeletal muscles. Our core objective was to reveal circRNA-associated ceRNA network so as to support further systematic studies of myogenesis.

## Results

### Muscle Fiber Type Distribution

**Fig. 1:**
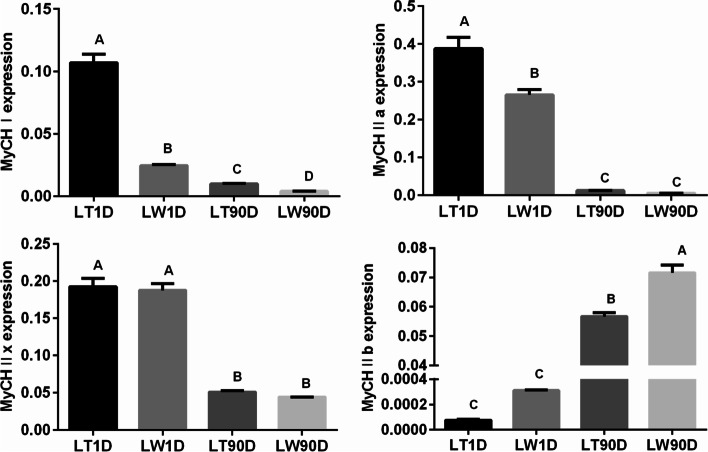
Relative expression level of MyHC isoforms (I, IIa, IIx, and IIb) in Lantang and Landrace pigs at birth and 90 postnatal days. Values represent mean ± SD of three biological replicates. LT1D, Lantang pig 1 day after birth; LW1D, Landrace pig 1 day after birth; LT90D, Lantang pig 90 days after birth; LW90D, Landrace pig 90 days after birth. Different superscripts indicate significant differences at *P* < 0.01

Hematoxylin and eosin staining was performed to characterize the structural traits of longissimus thoracis between Lantang and Landrace pigs at birth (LT1D and LW1D, respectively) and 90 postnatal days (LT90D and LW90D, respectively). The number of fibers per unit area and average cross-sectional area of myofibers were determined (Fig. S1). We found that the structural traits showed large variations between different growth stages (*P* < 0.01). To explain, the number of myofibers significantly decreased between birth and 90 postnatal days (*P* < 0.01), and the average cross-sectional area of myofibers showed an obvious increase during postnatal development (*P* < 0.01). Further, Lantang pigs showed higher number of myofibers than Landrace pigs at birth (*P* < 0.01), but there were no significant differences in terms of the cross-sectional area of myofibers. In comparison with Landrace pigs, Lantang pigs showed lower number and cross-sectional area of myofibers at 90 postnatal days (*P* < 0.01). We then calculated the proportion of different muscle fiber types based on the expression of myosin heavy chain isoforms (MyHCs; Fig. 1). The proportion of MyHC I, IIa, and IIx myofibers at birth was higher than that at 90 postnatal days in both Lantang and Landrace pigs (*P* < 0.01), while the proportion of MyHC IIb myofibers was higher at 90 postnatal days (*P* < 0.01). At birth, the expression of MyHC I and IIa in Lantang pigs was significantly higher than that in Landrace pigs (*P* < 0.01), while the expression of MyHC IIb was higher in Landrace pigs (*P* > 0.05). Besides, at 90 postnatal days, higher amount of MyHC I was distributed in Lantang pigs (*P* < 0.01), and MyHC IIb showed the opposite trend between Lantang and Landrace pigs (*P* < 0.01).

## miRNA Expression Analysis

miRNA-seq generated 21.06 ± 1.32 million raw reads with a length of 49 nucleotides from each library. After filtering, approximately 18.68 ± 1.33 million clean reads were obtained, accounting for 89.12% ± 3.47% of total raw sequences (Table S1A). The clean sequences were then annotated and assigned to 470.78 ± 86.52 thousand unique tags in each library by alignments to Rfam and porcine-specific sequences within miRBase, Repeat and Reference mRNA databases (Table S1B). We observed that only 3.16% ± 0.41% (11,464 ± 415 per library) unique reads belonged to known porcine miRNAs, and these unique reads represented 15.27% ± 0.69% (2,871,531 ± 269,401 per library) of total clean sequences. Using the miRDeep2 algorithm, 321, 311, 315, and 316 known miRNAs were identified in LT1D, LT90D, LW1D, and LW90D libraries, respectively, and 302 miRNAs were common across all samples. The 10 most highly expressed miRNAs in each library accounted for 71.26% ± 1.15% of the total count of all identified miRNAs, and six miRNAs (miR-1, miR-206, let-7a, let-7c, miR-10b, and let-7f) were found across all libraries. Of them, ssc-miR-206 showed the highest expression level in LT1D and LW1D libraries, as well as ssc-miR-1 in LT90D and LW90D libraries. We further investigated differentially expressed miRNAs between different breeds and growth stages (Table S1C). In Lantang pigs, 89 miRNAs were differentially expressed between LT1D and LT90D libraries; 34 miRNAs were upregulated and 55 were downregulated in LT90D libraries. In Landrace pigs, 100 differentially expressed miRNAs were identified; 19 miRNAs were upregulated and 81 were downregulated in LW90D libraries. At birth, only seven miRNAs were differentially expressed between LT1D and LW1D libraries, including six up- and one downregulated miRNAs in LW1D libraries, and at 90 postnatal days, 11 miRNAs were differentially expressed between LT90D and LW90D libraries, including four up- and seven downregulated miRNAs in LW90D libraries.

## Transcriptome Expression Analysis

**Fig. 2:**
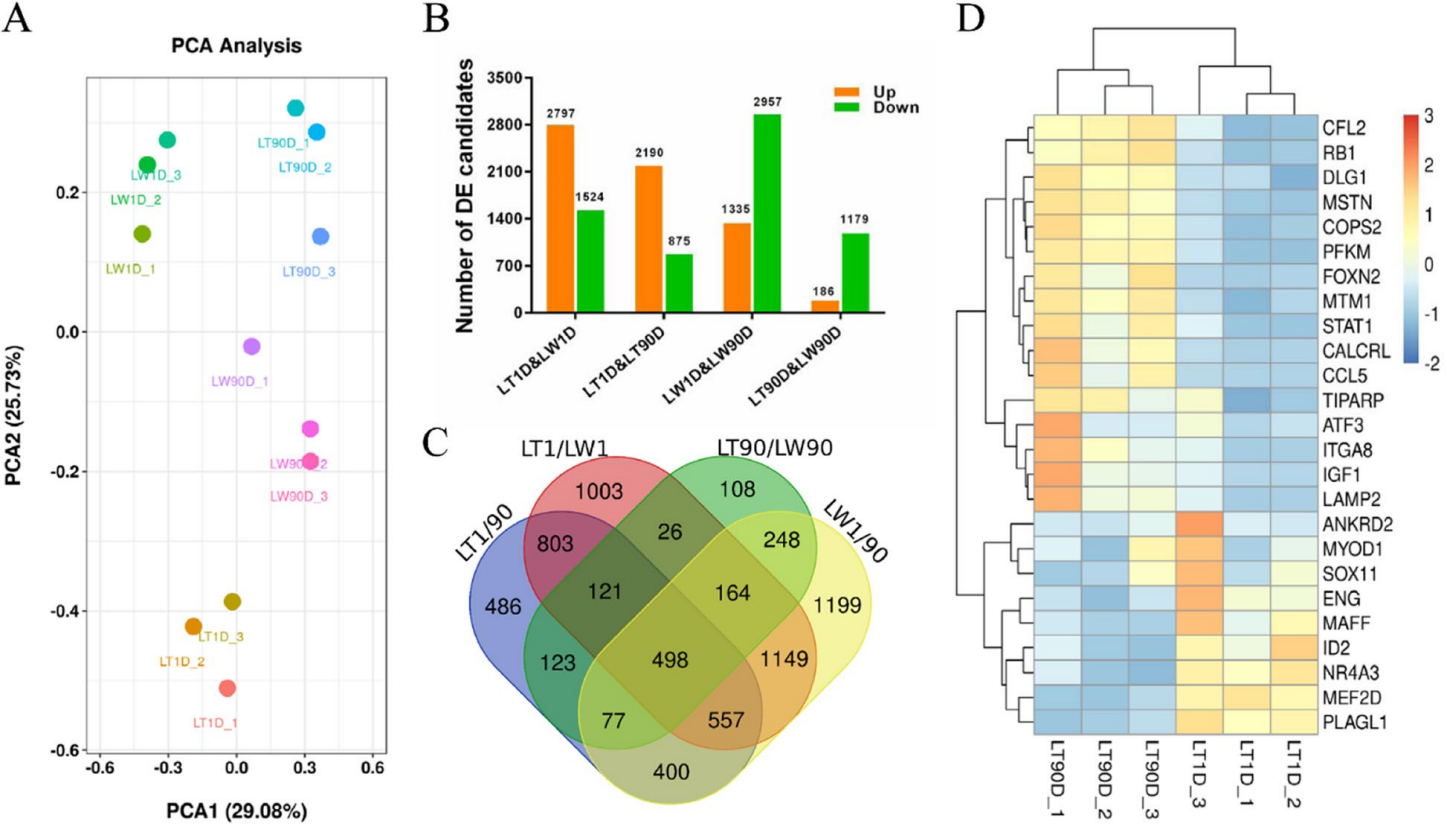
Transcriptome expression analyses of Lantang and Landrace pigs at birth and 90 postnatal days (**A**) Principal component analysis of globally expressed transcripts with FPKM levels. (**B**) Number of differentially expressed candidate transcripts between Lantang and Landrace at birth and 90 postnatal days. “Up” and “down” represent the number of transcripts with increased and decreased expression in the latter, respectively. (**C**) Common and unique differentially expressed candidate transcripts. (**D**) Heatmap showing differentially expressed transcripts significantly enriched in myogenesis (*P* < 0.05). Note: LT1D, Lantang pig 1 day after birth; LW1D, Landrace pig 1 day after birth; LT90D, Lantang pig 90 days after birth; LW90D, Landrace pig 90 days after birth

In total, 12 muscle tissue samples obtained from Lantang and Landrace pigs at birth and 90 postnatal days (in triplicate) were subjected to Illumina sequencing after rRNA depletion, which led to the generation of approximately 1.49 billion reads (average of 124.57 ± 0.29 million reads per sample). After quality control/trimming, 122.86 ± 0.21 million valid reads were obtained, accounting for 98.63% ± 0.19% of raw reads in each library. On alignment of all valid reads, we found that over 84.45% ± 1.46% clean reads could be successfully mapped to the porcine *Sscrofa11.1* reference genome, including 78.88% ± 1.26% mapped reads with proper pair alignment (Table S2A). Transcript assemblies with StringTie revealed 138,278 isoforms across the 12 libraries, including approximately 24.98% identified candidates that completely matched Ensembl transcript regions (Table S2B). A comparison of known Ensembl transcripts revealed that 39,734, 39,909, 40,445, and 38,429 known transcripts were expressed in LT1D, LT90D, LW1D, and LW90D libraries, respectively; 42,081 transcripts existed in all libraries (Table S3A). Principal component analysis of globally expressed transcripts with fragments per kilobase of transcript per million mapped reads (FPKM) levels was performed, which showed that the differences between groups caused by breed or age were much greater than those between experimental individuals (Fig. 2A). We therefore applied the Ballgown algorithm to analyze differences in libraries between different breeds and growth stages (Fig. 2B). With normalized RPKM, there were 4,321 differentially expressed Ensembl transcripts between LT1D and LW1D libraries; 2,797 transcripts were upregulated and 1,524 were downregulated in LW1D libraries (Table S3B). Between LT1D and LT90D libraries, we detected 3,065 differentially expressed transcripts; 2,190 transcripts were upregulated and 875 were downregulated in LT90D libraries (Table S3C). Further, 4,292 differentially expressed transcripts were identified between LW1D and LW90D libraries; 1,335 transcripts were significantly upregulated and 2,957 were downregulated in LW90D libraries (Table S3D). In comparison with LW90D libraries, the expression levels of 1,365 transcripts were significantly different in LT90D libraries; 186 and 1,179 transcripts were up- and downregulated in LW90D libraries, respectively (Table S3E). In total, 6,962 unique differentially expressed transcripts were found on comparing LT1/90D, LW1/90D, LT/LW90D, and LT/LW1D, and only 498 transcripts were common (Fig. 2C). Gene ontology (GO) analysis revealed that these differentially expressed transcripts were significantly enriched (*P* < 0.05) in several biological processes associated with myogenesis, including skeletal muscle cell differentiation, muscle cell cellular homeostasis, positive regulation of smooth muscle cell proliferation, smooth muscle tissue development, muscle contraction, regulation of skeletal muscle satellite cell proliferation, and response to muscle stretch (Table S4A–D), and 25 myogenesis-related transcripts were identified between LT1D and LT90D samples (Fig. 2D). Moreover, Kyoto Encyclopedia of Genes and Genomes (KEGG) pathway analysis revealed that several differentially expressed transcripts were involved in muscle development and growth pathways, such as mTOR signaling pathway, Wnt signaling pathway, AMPK signaling pathway, and biosynthesis of amino acids (Table S4E–H). We randomly selected 10 dysregulated mRNAs (PFKM, ANKRD2, MSTN, MYOD1, SRF, IGF1, MYBPC2, LIMCH1, PFKFB1, and MEF2D; Fig. S2A) from these myogenesis-related GO terms and signaling pathways and validated their expression levels by performing quantitative real-time PCR (RT-qPCR). Between LT1D and LW1D, RT-qPCR data revealed that the expression levels of ANKRD2, MYOD1, LIMCH1, and MEF2D were significantly upregulated in LT1D, but those of PFKM, MSTN, MYBPC2, SRF, and PFKEB1 did not show a significant change. The RT-qPCR results of MSTN, ANKRD2, and SRF were inconsistent with those of RNA-seq. According to RNA-seq data, there was no significant difference in the expression level of ANKRD2, whereas the expression levels of SRF and MSTN were significantly upregulated in LT1D and LW1D, respectively. Further, in the comparison between LT1D and LT90D, the expression levels of ANKRD2, MYOD1, LIMCH1, and MEF2D were significantly upregulated in LT1D, and those of PFKM, MSTN, SRF, and MYBPC2 were significantly upregulated in LT90D; PFKFB1 was not significantly differentially expressed. RNA-seq did not reveal any significant differences in LIMCH1 expression between LT1D and LT90D. In the comparison between LW1D and LW90D, the expression levels of ANKRD2 and MEF2D were significantly upregulated in LT90D, and those of MSTN, SRF, MYBPC2, and PFKFB1 were significantly upregulated in LW90D; PFKM, MYOD1, and LIMCH1 expression showed no significant differences. According to RNA-seq data, the expression level of SRF was significantly upregulated in LW1D, which contradicted RT-qPCR results. In the comparison between LT90D and LW90D, the expression level of PFKM was significantly upregulated in LT90D and that of PFKFB1 was significantly upregulated in LW90D, but MSTN, MYOD1, MYBPC2, LIMCH1, ANKRD2, and SRF expression levels showed no significant differences. The results for MSTN, PFKFB1, and PFKM were inconsistent between RT-qPCR and RNA-seq. RNA-seq data indicated that the expression levels of PFKFB1 and PFKM did no show a significant change between LT90D and LW90D, while the expression level of MSTN was significantly upregulated in LW90D (Fig. S2B).

## Identification of circRNAs

**Fig. 3:**
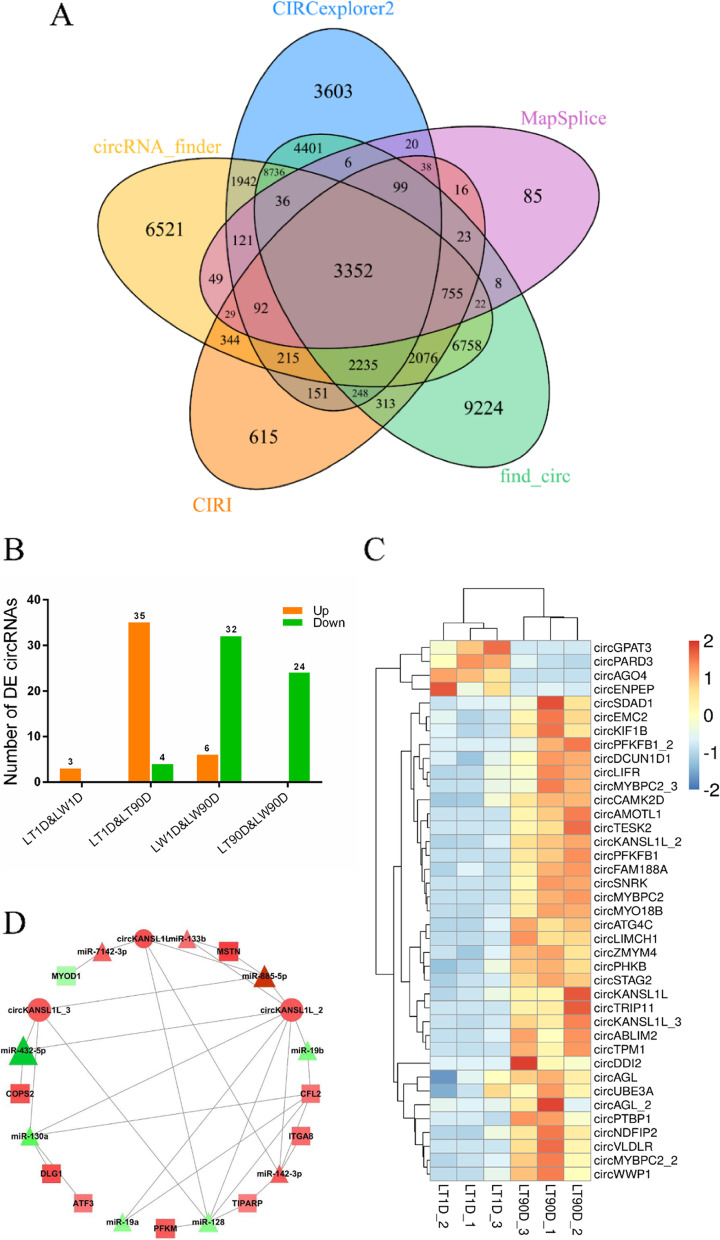
Identification of circRNA candidates using five predicting algorithms (**A**) Common circRNA candidates identified by all five algorithms. (**B**) Number of differentially expressed circRNAs between Lantang and Landrace pigs at birth and 90 postnatal days. “Up” and “down” represent the number of circRNAs with increased and decreased expression in the latter, respectively. (**C**) Heatmap showing differentially expressed circRNAs. (**D**) 31 up–down–up regulation patterns of circRNA-miRNA-mRNA correlation networks between LT1D and LT90D comparison. Solid circles represent circRNAs, triangles represent miRNAs, and squares represent coding genes. Red represents upregulation and green represents downregulation in Lantang pigs. Color depth represents –log(FDR value, 2), and a darker color represents greater significant. Size represents log(mean FPKM level in LT1D, 10), and a bigger size represents greater expression in Lantang pigs at birth. Note: LT1D, Lantang pig 1 day after birth; LW1D, Landrace pig 1 day after birth; LT90D, Lantang pig 90 days after birth; LW90D, Landrace pig 90 days after birth

We characterized circRNA landscape and expression by performing deep RNA-seq experiments using the 12 aforementioned muscle tissue samples. In total, 52,133 circRNA candidates were identified using five different predicting algorithms (Fig. 3A); circRNA landscape differed quite radically depending on the algorithm used. To explain, 25,295, 33,283, 10,601, 38,292, and 4,751 circRNA candidates were detected by CIRCexplorer2, circRNA_Finder, CIRI, find_circ, and MapSplice algorithms, respectively (Table S5); find_circ and MapSplice exhibited the highest and lowest level of sensitivity, respectively. Only 3,352 circRNA candidates were commonly detected by all five algorithms, and these were subjected to further analyses. These circularization events were found to be produced from 1,745 hosting transcript loci, including 712 transcripts that generated multiple circRNA candidates (Table S6). With normalized back-splice junction reads, we analyzed significant differences in circRNA candidates across four comparisons: LT/LW1D, LT1/90D, LW1/90D, and LT/LW90D (Fig. 3B). Only three differentially expressed circRNA candidates were found between LT1D and LW1D libraries, and all three of them were significantly upregulated in LW1D library. Further, 39 and 38 circRNA candidates were differentially expressed between LT1D and LT90D libraries and between LW1D and LW90D libraries, respectively (Fig. 3C). Interestingly, 24 differentially expressed circRNA candidates were differentially expressed between LT90D and LW90D libraries, and all of them were downregulated in LW90D libraries.

## Construction of circRNA-associated-ceRNA Networks

The expression of circRNAs potentially plays a key role in physiological and pathological conditions by regulating endogenous RNA targets [25]. We therefore performed Pearson correlation analysis to assess the association between differentially expressed circRNAs and mRNAs in each comparison (Fig. S3), which revealed 8, 187, 456, and 69 significant interactions in LT1D vs. LW1D, LT1D vs. LT90D, LW1D vs. LW90D, and LT90D vs. LW90D, respectively. Few circRNA candidates have been reported to directly modulate the transcription of their parent genes [26]. Herein we found that only circANKRD2, derived from exons 3 and 4 of *ANKRD2*, was positively correlated with its linear counterpart at the expression level between LW1D and LW90D, suggesting the involvement of circANKRD2 and *ANKRD2* in myogenesis. In addition, it has been found that endogenous circRNAs serve as miRNA sponges to consequently repress the function of their targets [27]. This prompted us to predict shared miRNA-binding sites between differentially expressed circRNAs and mRNAs (Table S7A–G) and further analyze circRNA-miRNA-mRNA ceRNA networks. We identified 777, 855, and 22 convincing ceRNA interactions in LT1D vs. LT90D, LW1D vs. LW90D, and LT90D vs. LW90D, respectively (Table S7H–J); the number of putative interactions per miRNA markedly varied, ranging from 1 to 51 miRNA-associated ceRNA networks. We observed that the highly expressed circKANSL1L, circKANSL1L_2, circKANSL1L_3, circKANSL1L_4, and circKANSL1L_5 participated in 279 ceRNA transcriptional regulatory axes, including a total of 27 unique myo-miRNAs and 30 special myogenes. As evident from Fig. 3D, LT1D and LT90D comparison revealed 31 up–down–up regulation patterns: circKANSL1L, circKANSL1L_2, and circKANSL1L_3 were upregulated in LT90D and could sponge miR-128, miR-130a, miR-133b, miR-142-3p, miR-19a, miR-19b, miR-432-5p, miR-7142-3p, and miR-885-5p to significantly upregulate *ATF3*, *CFL2*, *COPS2*, *DLG1*, *ITGA8*, *MSTN*, *MYOD1*, *PFKM*, and *TIPARP* expression (Table S7H). On the contrary, 14 down–up–down regulation patterns were identified on comparing LW1D and LW90D: circKANSL1L_4 was downregulated in LW90D and could sponge miR-130a, miR-19a, miR-19b, miR-299, miR-376a-3p, miR-487b, and miR-493-5p to significantly downregulate *ACTN2*, *COPS2*, *FBXO40*, *FOXN2*, *MYBPC1*, *SCN7A*, *TPM3*, and *TPM4* expression (Table S7I).

## Characterization of Myogenesis-related circRNAs

To verify the circular structure of circRNAs, differentially and highly expressed circRNA candidates that were correlated with myogenes were selected for further analyses. cDNA was amplified using a pair of divergent primers, which led to the identification of nine circRNAs: circPFKFB1, circKANSL1L-3, circLIMCH1, circKANSL1L, circ4082, circKANSL1L-2, circMYBPC2, circMYBPC2-2, and circNR1H3 (Fig. S4A). Sanger sequencing further verified their head–tail junction structure (Fig. S4B).

**Fig. 4:**
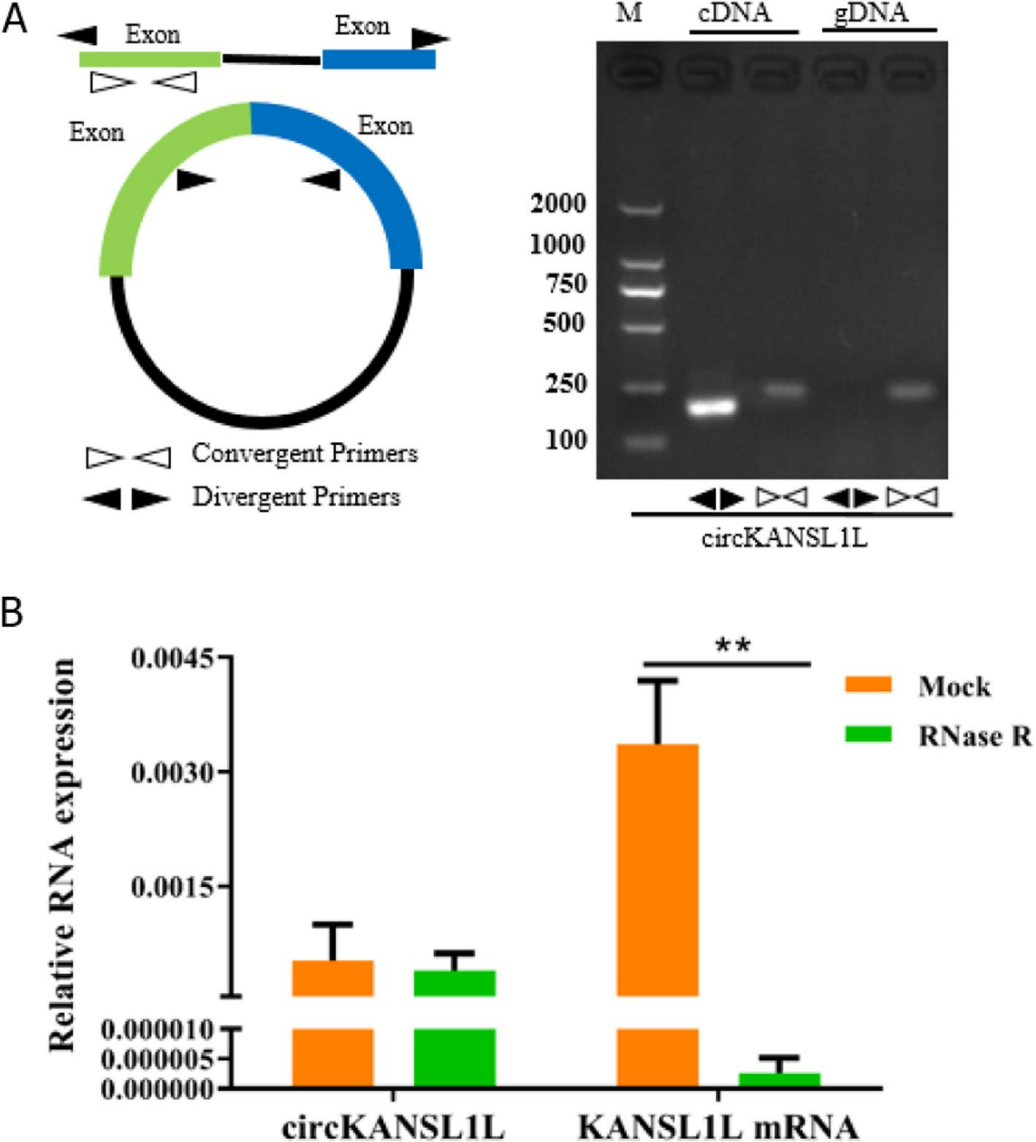
Verification of circRNA structure (**A**) Identification of circRNA candidates using divergent and convergent primers. Divergent primers amplified circRNA targets in cDNA but not genomic DNA, and convergent primers amplified targets in both. (**B**) RT-qPCR to assess circRNA abundance and mRNA expression of host genes. *GAPDH* served as the reference gene. Note: Values are mean ± S.E.M. Data represent six independent assessment methods. Student’s *t*-test was used to compare expression levels or values among different groups. *P < 0.05; **P < 0.01

Furthermore, on comparing the homology of the nine aforementioned circRNAs, we found that circKANSL1L sequence showed high homology between mice and pigs based on NCBI blastn suite (Fig. S5). To further verify their structure, we analyzed them in C_2_C_12_ cells using divergent and convergent primers (Fig. 4). Convergent primers could successfully amplify both cDNA and genomic DNA, but divergent primers could only amplify cDNA (Fig. 4A). On RNase R digestion and RT-qPCR of the circRNAs and hosting mRNA, we found that there was no significant change in circRNA expression levels between the RNase R treatment and control groups. However, the mRNA expression level of the hosting gene was significantly different (*P* < 0.01, Fig. 4B). These findings further suggested that the structure of circKANSL1L was indeed circular.

## Inhibition of C_2_C_12_ Cell Proliferation by circKANSL1L

**Fig. 5:**
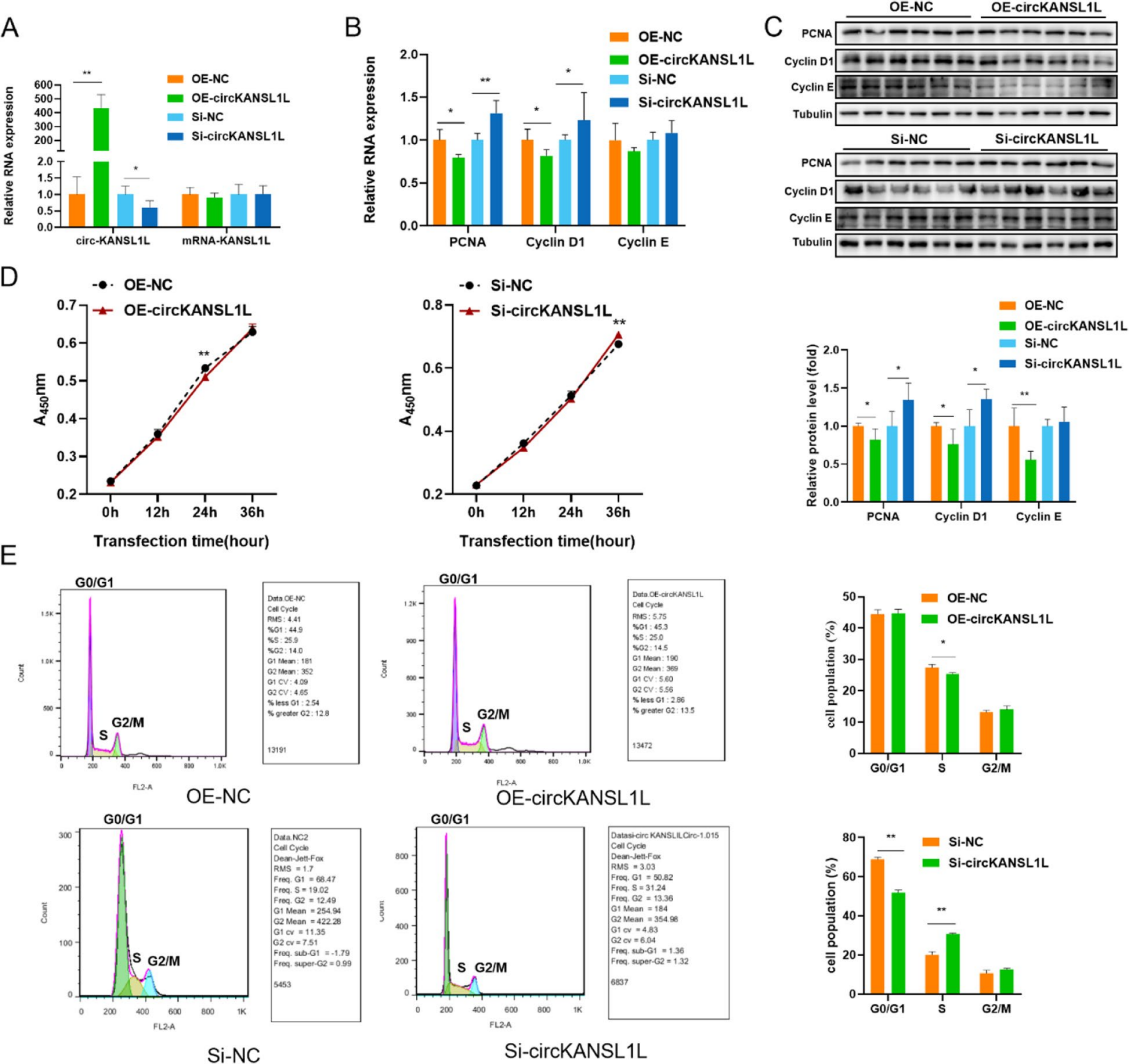
Inhibition of C_2_C_12_ cell proliferation by circKANSL1L (**A**) Relative expression of circKANSL1L and mRNA-KANSL1L in C_2_C_12_ cells following circKANSL1L overexpression or inhibition. *GAPDH* served as the reference gene. (**B**) Relative RNA and (c, above panel) protein levels of cell proliferation-related genes in C_2_C_12_ cells following circKANSL1L overexpression or inhibition. (**C**, below panel) PCNA, CyclinD1, and CyclinE band intensities quantified by Image J and normalized against internal reference Tubulin. (**D**) Growth curve of C_2_C_12_ cells following circKANSL1L overexpression and inhibition. (**E**) Cell cycle analysis of C_2_C_12_ cells following circKANSL1L overexpression or inhibition. Note: Values are mean ± S.E.M. Data represent six independent assessment methods. Student’s *t*-test was used to compare expression levels or values among different groups. *P < 0.05; **P < 0.01

To elucidate the role of circKANSL1L in myogenesis, we constructed the overexpression plasmid OE-circKANSL1L and designed the knockdown gene si-circKANSL1L, and RT-qPCR was performed to verify their effects. We found that OE-circKANSL1L and si-circKANSL1L significantly increased and decreased the expression of circKANSL1L in C_2_C_12_ cells, but the expression of the host gene KANSL1L was unaffected (Fig. 5A).

Besides, RT-qPCR was performed to assess the relative expression levels of the cell proliferation-related genes PCNA, Cyclin D1, and Cyclin E. After circKANSL1L overexpression, the expression level of PCNA (*P* < 0.05) and Cyclin D1 (*P* < 0.05) significantly decreased in C_2_C_12_ cells and that of Cyclin E showed the same trend (*P* > 0.05). After circKANSL1L knockdown, the expression level of PCNA (*P* < 0.01) and Cyclin D1 (*P* < 0.05) significantly increased in C_2_C_12_ cells and that of Cyclin E also increased, but the change was not significant (*P* > 0.05; Fig. 5B). When circKANSL1L expression was upregulated, the protein expression levels of PCNA (*P* < 0.05), Cyclin D1 (*P* < 0.01), and Cyclin E (*P* < 0.01) significantly decreased, and when circKANSL1L expression was downregulated, the protein expression levels of PCNA (*P* < 0.05) and Cyclin D1 (*P* < 0.05) significantly increased and that of Cyclin E also increased, but the change was not significant (*P* > 0.05; Fig. 5C), these results were consistent with RT-qPCR results. On transfecting C_2_C_12_ cells with empty vector and OE-circKANSL1L, cell proliferation was measured by Cell Counting Kit-8 (CCK-8) assay at 0, 12, 24, and 36 h. In comparison with the empty vector group, after circKANSL1L overexpression, absorbance (450 nm) significantly decreased at 24 h (*P* < 0.01); however, after circKANSL1L knockdown, absorbance (450 nm) significantly increased at 36 h (*P* < 0.01; Fig. 5D). In addition, our cell cycle analysis showed that when circKANSL1L was overexpressed, cells were arrested in the G1 phase, and the number of cells entering the S phase was significantly lower than that in the control group (*P* < 0.05). circKANSL1L knockdown promoted the progression of C_2_C_12_ cells to the S and G2 phases (*P* < 0.01, Fig. 5E). Altogether, these results indicated that circKANSL1L inhibited the proliferation of C_2_C_12_ cells.

## Enhancement of C_2_C_12_ Cell Differentiation by circKANSL1L

**Fig. 6:**
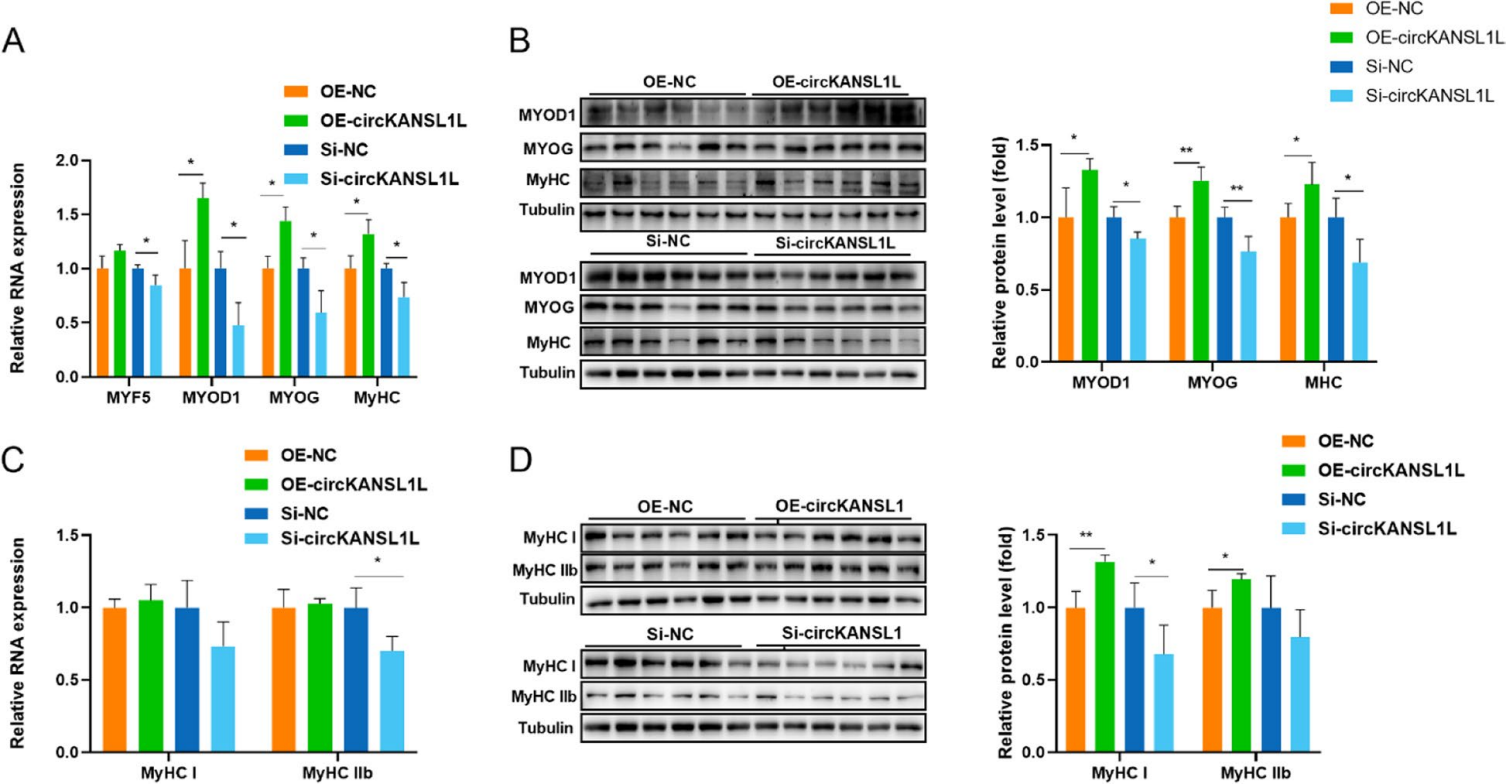
Enhancement of C_2_C_12_ cell differentiation by circKANSL1L (**A**) Relative RNA levels of muscle cell differentiation marker genes in C_2_C_12_ cells following circKANSL1L overexpression or inhibition. *GAPDH* served as the reference gene. (**B**) Protein levels of MYOG and MyHC in C_2_C_12_ cells following circKANSL1L overexpression or inhibition (left panels). MYOG and MyHC band intensities were quantified by Image J and normalized against GAPDH (right panels). (**C**) Relative RNA levels of MyHC I and IIb in C_2_C_12_ cells following circKANSL1L overexpression or inhibition. (**D**) Protein levels of MyHC I and IIb in C_2_C_12_ cells following circKANSL1L overexpression or inhibition (left panels). MyHC I and IIb band intensities were quantified by Image J and normalized against GAPDH (right panels). Values are mean ± S.E.M. Data represent six independent assessment methods. Student’s *t*-test was used to compare expression levels or values among different groups. *P < 0.05; **P < 0.01

RT-qPCR was performed to assess the relative expression levels of MYF5, MYOD1, Myogenin (MYOG), and MyHC (Fig. 6A). circKANSL1L overexpression significantly increased the expression levels of MYF5 (*P* = 0.06), MYOD1 (*P* < 0.05), MYOG (*P* < 0.05), and MyHC (*P* < 0.05), while circKANSL1L knockdown significantly decreased their expression levels (*P* < 0.05 for all). Western blotting was performed to detect MYOD1, MYOG and MyHC protein expression levels (Fig. 6B). The results showed the same trend as RT-qPCR results, and the data were significant (MYOD1, *P* < 0.05; MYOG, *P* < 0.01; MyHC, *P* < 0.05). We also assessed RNA expression levels of MyHC I and MyHC IIb. CircKANSL1L overexpression promoted MyHC I and MyHC IIb expression, while circKANSL1L knockdown inhibited MyHC I and MyHC IIb expression, but the results were insignificant (Fig. 6C). Protein expression levels showed a consistent trend with RNA expression levels (Fig. 6D). When circKANSL1L expression was upregulated, the protein expression levels of MyHC I (*P* < 0.01) and MyHC IIb (*P* < 0.05) significantly increased, and when circKANSL1L expression was downregulated, the protein expression levels of MyHC I (*P* < 0.05) significantly decreased and MyHC IIb decreased but no significantly (*P* > 0.05), indicating that circKANSL1L has a regulatory effect on muscle fiber type differentiation; further studies are nevertheless warranted.

## Discussion

The demand for better meat quality is exponentially increasing among consumers each year, with tenderness, color, water-holding capacity, and flavor of meat being key factors [28]. However, the long-term artificial selection for growth rate and lean meat rate has led to the deterioration of meat quality [29, 30]. Accordingly, several studies focusing on effective strategies to improve meat quality have been reported. Muscle fiber type is evidently a pivotal factor affecting meat quality [31], as it influences the color, water-holding capacity, tenderness, and flavor of meat [32]. Muscle fibers occupy 75%–90% of the total muscle volume [33]. According to the contractile and metabolic properties and morphological traits of muscles, muscle fibers can be differentiated into oxidative and glycolytic types [9]. The proportions of these different muscle fibers reportedly affect meat quality [34]. Larzul et al*.* (1997) reported that a decrease in the proportion of glycolytic fiber is beneficial to meat tenderness, color, and water-holding capacity [35]. However, it is notable that various factors influence the proportion and distribution of muscle fiber types [33], such as breed, genotype, feeding, slaughter method, chilling, and storage conditions [7, 8].

Landrace pigs (lean type pigs) and Lantang pigs (obese type pigs) have great differences in meat quality, such as fat content, meat color and tenderness [36, 37]. Therefore, in this study, longissimus thoracis muscle tissues were collected from Landrace pigs and Lantang pigs at birth and 90 postnatal days. Through hematoxylin and eosin staining and RT-qPCR, we found that species as well as age affected the distribution of muscle fiber types. There were considerable differences in oxidative and glycolytic muscle tissues between different species and growth stages, and this finding was consistent with that of Zhao et al*.*[38]. Therefore, these two types of porcine muscle tissues can be reliably used to comprehend the mechanism underlying muscle development and phenotypic differences [40]. In addition, RNA-seq analysis revealed that some differentially expressed genes between Lantang and Landrace pigs at birth and 90 postnatal days were significantly enriched in myogenesis, and RT-qPCR revealed a small but specific set of these differentially expressed genes with inconsistent expression measurements to RNA-seq analysis. In general, these genes were typically lower expressed, smaller and had fewer exons [41].

With the development of sequencing technology, an increasing number of studies have indicated that ncRNAs play a regulatory role in myogenesis [40, 42, 43]. circRNAs are a type of covalently closed circular RNAs [14], and miRNAs are a class of short ncRNAs with a length of approximately 22 bp [43]. Although the functions of circRNAs remain largely unexplored, they serve as miRNA sponges [24] and ultimately affect mRNA expression [44]. For example, circLMO7 regulates the expression of HDAC4 mRNA by adsorbing miR-378a-3p [27], thereby inhibiting myogenic differentiation. circFRFR4 binds to miR-107 to competitively regulate Wnt3a expression and promote bovine myoblast differentiation [18], and circFUT10 directly binds to mir-133a to regulate myoblast differentiation [45]. Moreover, circHUWE1 targets AKT3 by adsorbing mir-29b, consequently promoting myoblast proliferation and inhibiting cell differentiation [46], and circZFP609 can sponge mir-194-5p to sequester its inhibition on BCLAF1 so as to repress myogenic differentiation [47]. Further, circHIPK3 has been reported to regulate myoblast proliferation and differentiation through the miR-7/TCF12 pathway [48]. Some studies have also shown that circRNAs, such as circZNF609 [49] and circFAM188B [50], play a role in myogenesis directly by translating proteins. Therefore, we explored circRNA transcriptome using oxidative and glycolytic muscle tissues obtained from pigs of different growth stages and revealed circRNA-associated ceRNA networks for further systematic studies of myogenesis. Our experiments led to the identification of differentially expressed miRNAs, mRNAs, and circRNAs between different pig breeds and growth stages. By GO and KEGG pathway analyses, we established a potential molecular signaling pathway for differentially expressed mRNAs, which was related to muscle development. Next, we performed Pearson correlation analysis to study the interaction between circRNAs and mRNA, and finally, a circRNA-miRNA-mRNA ceRNA network related to muscle growth and development was identified. We noticed that circKANSL1L, which was differentially expressed in Lantang pigs at birth and 90 postnatal days, showed a high expression level and participated in pairs of circRNA-miRNA-mRNA networks. It seems that these network pairs play a crucial role in myogenesis. Altogether, our findings provide a new direction for studying muscle formation and a theoretical basis for improving meat quality.

In general, PCNA is involved in DNA synthesis and DNA damage repair [51], and Cyclin D1 and Cyclin E are regulatory factors of cell cycle progression [52]; all of them reflect the growth rate and state of cells. Our results showed that circKANSL1L decreased the expression level of PCNA, Cyclin D1, and Cyclin E in C_2_C_12_ cells, eventually inhibiting their proliferation. Elnour et al*.* also used PCNA and Cyclin D1 as marker genes to evaluate the state of cell proliferation, and they found that circMYL1 inhibited the proliferation of bovine primary myoblasts by sponging miR-2400 [53]. Our cell cycle analysis and CCK-8 assay results also indicated that circKANSL1L decreased the proliferation rate of C_2_C_12_ cells. Li et al*.* found that circFUT10 inhibited myoblast proliferation by blocking cells in the G1/G0 phase [45]. Myf5, MyoD1, and MyoG, as core myogenic regulators, play a key role in myogenesis [54, 55]. The transcription factor MYOG is involved in the regulation of myocyte fusion and is essential for the growth of muscle fibers and proliferation of muscle nuclei [56]. MyHC is the basic unit of myosin, and its expression level indicates the differentiation process of myoblasts [57]. In the present study, circKANSL1L overexpression was found to enhance RNA expression levels of MYF5, MYOD1, MYOG, and MyHC. At the same time, protein expression levels of MYOG and MyHC were detected using Western blotting, and the obtained results showed the same trend as RNA expression levels, further confirming that circKANSL1L promoted the differentiation of C_2_C_12_ cells. Several studies have reported that circRNAs are involved in muscle fiber differentiation. Shen et al*.* determined the expression levels of MYOD1, MYOG, and MyHC to report that circTMTC1 inhibited the differentiation of chicken skeletal muscle satellite cells into myotubes by sponging miR-128-3p [58]. Ouyang et al*.* found that circSVIL overexpression upregulated the mRNA and protein levels of MYOG and MHC, suggesting that circSVIL promoted myoblast differentiation [59]. To summarize, an increasing number of circRNAs are being reported to play a biological role in myogenesis, and our results should serve as a reference.

## Conclusions

To conclude, RNA-seq was performed to identify genome-wide circRNAs in 0- and 90-day-old Lantang pigs and Landrace pigs, which revealed that circRNAs were abundant, differentially expressed, and involved in myogenesis. We also identified a novel circRNA, circKANSL1L, which was found to inhibit the proliferation of C_2_C_12_ cells but promote their differentiation.

## Methods

### Tissue Preparation

Landrace (lean type) and Lantang (fat type) pigs were obtained from Banling breeding farm (Xinfeng County, Shaoguan City, Guangdong Province, China). Five body weight- and sex-balanced piglets of each breed were humanely slaughtered at birth and at 3 months of age (i.e., 10 pigs of each breed); subsequently, 20 longissimus thoracis muscle tissues were immediately collected and snap-frozen in liquid nitrogen for further analyses. In addition, any anesthesia or euthanizing agent was not used in our study.

## Muscle Fiber Characteristics

After carcass bleeding, a part of the muscle tissue was cut into approximately 0.5 × 0.5 × 1.0 cm pieces, which were then immediately fixed in 4% paraformaldehyde for 24 h. The samples were then immersed in xylene–alcohol (1:1, v/v), infiltrated, and embedded in paraffin. Cross-sections were prepared at 3-μm thickness, stained with hematoxylin and eosin, viewed under a microscope, and photographed (200 × and 400 × magnification). The number of myofibers and total cross-sectional areas were subsequently assessed using Image-Pro Plus v6.0 (Media Cybernetics Inc., Rockville, MD, USA). In addition, relative expression levels of MyHC isoforms (I, IIa, IIx, and IIb) were analyzed by RT-qPCR, with *GAPDH* serving as the reference gene.

## Library Preparation and RNA-seq

Total RNA was extracted from the tissue samples using TRIzol (Takara, Dalian, China). RNA quantity and purity were determined using an Agilent 2100 Bioanalyzer and the RNA 6000 Nano LabChip Kit (Agilent, Santa Clara, USA). For miRNA-seq library construction, RNA fragments of 18–30 nucleotides were separated and enriched by 15% polyacrylamide gel electrophoresis, and proprietary indexed adapters were then ligated to 5ʹ- and 3ʹ-termini. Subsequently, reverse transcription was performed, followed by low-cycle PCR, to obtain sufficient products for Illumina sequencing. For RNA-seq library construction, approximately 10 μg of total RNA per sample was used to deplete rRNA, according to the instructions of the Epicentre Ribo-Zero Gold Kit (Illumina, San Diego, USA), which was followed by TRIzol extraction. The rRNA-depleted RNAs were then fragmented and reverse-transcribed to obtain cDNA libraries using the RNA Library Prep Kit (Illumina). Finally, paired-end sequencing was performed on an Illumina Hiseq4000 platform (LC Sciences, Hangzhou, China).

## Primary Analysis

We first used FastQC v0.11.9 (http://www.bioinformatics.babraham.ac.uk/projects/fastqc/) to evaluate the preliminary quality of raw sequences and then Cutadapt v2.6 [60] to filter low quality reads and bases contaminated with adapters. Using the SOAP algorithm [61], filtered reads from miRNA-seq libraries were aligned and annotated against porcine mRNA (ftp://ftp.ensembl.org/pub/release-96/fasta/sus_scrofa/cdna/) and CDS (ftp://ftp.ensembl.org/pub/release-96/fasta/sus_scrofa/cds/), Rfam v14.2 (http://rfam.xfam.org/), RepeatMasker (http://www.repeatmasker.org), and miRBase v22.1 (http://www.mirbase.org/). The types and abundance distribution of known porcine miRNAs were further analyzed and counted using miRDeep2 package v2.0.0.8 with the Perl script ‘quantifier.pl’ [62]. The edgeR package v3.30.3 (https://bioconductor.org/packages/edgeR/) [63] was then used to identify differentially expressed miRNAs with FDR < 0.05. In addition, clean reads from RNA-seq libraries were mapped to the *Sscrofa11.1* reference genome (ftp://ftp.ensembl.org/pub/release-94/fasta/sus_scrofa/dna/) using HISAT2 v2.1.0, and StringTie v2.0.6 was used to assemble and quantify transcripts in each library [64]. mRNA expression levels were measured and normalized as FPKM, and Ballgown v2.20.0 [64] was used to identify and compare differentially expressed transcripts and produce tables and plots. To predict circRNA candidates, we used five different algorithms: CIRCexplorer2 [65], circRNA_Finder [66], CIRI [67], find_circ [68], and MapSplice [69]. Only circRNA candidates that were identified by all of them were further analyzed. The expression levels of circRNA candidates were calculated with back-splice junction reads, and the edgeR algorithm was applied to examine their differential expression (FDR < 0.05). Finally, biological processes (GO terms) and KEGG pathway analyses [70] were performed using DAVID (https://david.ncifcrf.gov/).

## Cell Culture

The mouse myoblast cell line C_2_C_12_ was purchased from American Type Culture Collection. The cells were grown in a growth medium [GM, Dulbecco’s modified Eagle’s medium (DMEM; Gibco, Grand Island, NY, United States) + 10% fetal bovine serum (Gibco) + 1% penicillin–streptomycin (Invitrogen, Carlsbad, CA, United States)] and induced to differentiate in a differentiation medium [DM, DMEM + 2% horse serum (Gibco) + 1% penicillin–streptomycin (Invitrogen)] when they reached 90% confluence. The cells were cultured in a humidified incubator at 37 °C and 5% CO_2_.

## RT-qPCR

Total RNA was isolated from the muscle tissue samples and C_2_C_12_ cells using TRIzol, and cDNA was synthesized from RNA using the PrimeScript™ RT Reagent Kit with gDNA Eraser (Takara), according to manufacturer instructions. Genomic DNA was extracted from C_2_C_12_ cells using a kit (Sangon, Shanghai, China). To verify the circular structure of circRNAs, we designed a pair of convergent and divergent primers and verified their head-to-tail splicing using PCR and Sanger sequencing (Sangon). The primer sequences used in the experiment are listed in Table S8. Moreover, 2 µg total RNA from C_2_C_12_ cells was incubated with 3 U/µL ribonuclease R (RNase R) at 37 °C for 10 min; total RNA without RNase R (i.e., mock control) was also incubated under the same conditions. Gene expression levels were determined using the 2^−△△CT^ method. *GAPDH* served as the reference gene.

## Vector Construction and RNA Oligonucleotides

To synthesize the full-length linear sequence of circKANSL1L, a primer was designed using Primer 5.0. This sequence was amplified using C_2_C_12_ cDNA and subsequently cloned into pCD2.1-ciR (Geneseed Biotech, Guangzhou, China) using the KpnI and BamHI (Takara) restriction sites (OE-circKANSL1L). The empty vector was used as the negative control (OE-NC). siRNAs targeting circKANSL1L junction sites (si-circKANSL1L) and negative control (si-NC) were designed and synthesized by GenePharma Co., Ltd. (Shanghai, China).

## Transfection

C_2_C_12_ cells were transfected with OE-circKANSL1L, OE-NC, si-circKANSL1L, and si-NC using Lipofectamine 2000 (Invitrogen, Carlsbad, CA), according to manufacturer instructions, when they reached approximately 60% confluence. si-RNA transfection mix (20 pmol si-circKANSL1L or si-NC + 50 µL serum-free DMEM) or plasmid DNA transfection mix (1 µg OE-circKANSL1L or 1 µg OE-NC plasmid DNA + 50 µL serum-free DMEM) was prepared for each well, incubated at room temperature for 20 min, and subsequently diluted with transfection medium (1 μL lip 2000 + 50 μL serum-free DMEM). This mix was then added to each well, and the medium was replaced to GM after 6 h. The cells were harvested for protein and RNA analyses after 48 h to study cell proliferation. Further, the medium was switched to DM after 48 h, and the cells were collected for protein and RNA analyses at 96 h to study cell differentiation.

## CCK-8 Assay

CCK-8 (EZBioscience, Roseville, MN) assay was used to evaluate cell proliferation. Approximately 10^4^ cells were seeded in 96-well plates. After they adhered to the wall, they were transfected with OE-circKANSL1L, OE-NC, si-circKANSL1L, or si-NC. Six hours after transfection was recorded as 0 h. CCK-8 was added at 0, 12, 24, and 36 h, followed by incubation for 1 h. Absorbance was then measured at 450 nm using a microplate reader (Thermo Fisher Scientific, Waltham, MA).

## Flow Cytometric Cell Cycle Analysis

C_2_C_12_ cells were transfected with OE-circKANSL1L, OE-NC, si-circKANSL1L, and si-NC. After 48 h, the cells were collected, fixed with 75% ethanol, and stored overnight at − 20 °C. They were then resuspended in 500 μL PI/RNase staining buffer solution (BD Biosciences, Franklin Lakes, NJ) and incubated at 37 °C for 30 min. A BD Accuri C6 flow cytometer and FACSDiVa software (BD Biosciences) were used to perform flow cytometric analysis.

## Western Blotting

The cells were lysed using RIPA lysis buffer (Solarbio Life Sciences, Beijing, China) to obtain proteins, which were then separated by 10% sodium dodecyl sulfate–polyacrylamide gel electrophoresis, transferred to a 0.45-mm polyvinylidene fluoride membrane (Sigma, St. Louis, MO), and sealed with 5% skim milk for 2 h at room temperature. The cells transfected for 48 h were incubated overnight at 4 °C with the following primary antibodies: PCNA, Cyclin D1, Cyclin E, and Tubulin (ZenBio, Chengdu, China). Further, the cells transfected and differentiated for 96 h were incubated overnight with following primary antibodies: MYOG, MyHC, Tubulin (ZenBio), MyHC I, MyHC IIb, and GAPDH (ABclonal, Wuhan, China). After washing with Tris-buffered saline with Tween 20, the secondary antibody goat anti-rabbit IgG-HRP or goat anti-mouse IgG-HRP (Bioworld, Minneapolis, MN) was added, followed by incubation at room temperature for 1 h. Finally, enhanced chemiluminescence luminous fluid (Solarbio Life Sciences) was used for band visualization.

## Statistical Analysis

The comparative analysis of two groups was performed using unpaired independent *t*-test, and multiple comparative analysis was performed with one-way ANOVA. SPSS 20.0 (SPSS Inc., Chicago, IL) was used for statistical analyses. *P* < 0.05 and *P* < 0.01 indicated different and statistically different, respectively.

## Supplementary Information

**Additional file 1.** 12864_2022_8515_MOESM1_ESM.pdf.

**Additional file 2.** 12864_2022_8515_MOESM2_ESM.pdf.

**Additional file 3.** 12864_2022_8515_MOESM3_ESM.pdf.

**Additional file 4.** 12864_2022_8515_MOESM4_ESM.pdf.

**Additional file 5.** 12864_2022_8515_MOESM5_ESM.pdf.

**Additional file 6.** 12864_2022_8515_MOESM6_ESM.xlsx.

**Additional file 7.** 12864_2022_8515_MOESM7_ESM.xlsx.

**Additional file 8.** 12864_2022_8515_MOESM8_ESM.xlsx.

**Additional file 9.** 12864_2022_8515_MOESM9_ESM.xlsx.

**Additional file 10.** 12864_2022_8515_MOESM10_ESM.xlsx.

**Additional file 11.** 12864_2022_8515_MOESM11_ESM.xlsx.

**Additional file 12.** 12864_2022_8515_MOESM12_ESM.xlsx.

**Additional file 13.** 12864_2022_8515_MOESM13_ESM.xlsx.

## Data Availability

The raw sequences were deposited into Sequence Read Archive (SRA) database with the BioProject accession number PRJNA778795 (https://www.ncbi.nlm.nih.gov/bioproject/PRJNA778795/).

## Abbreviations

NcRNA: Noncoding RNAs
circRNAs: Circular RNAs
MyHCs: Myosin heavy chain isoforms
mTOR: Mammalian target of rapamycin
AMPK: Adenosine 5’-monophosphate-activated protein kinase
CDS: Coding sequence
DAVID: The Database for Annotation, Visualization and Integrated Discovery
KEGG: Kyoto Encyclopedia of Genes and Genomes
