# Supplementary material for: Identification of circRNA-associated ceRNA networks using longissimus thoracis of pigs of different breeds and growth stages
Source: BMC Genomics. 2022 Apr 11;23:294. doi: 10.1186/s12864-022-08515-7 (PMC9004053; doi:10.1186/s12864-022-08515-7)
Supplement: Supplementary file 1 — Additional file 1. 12864_2022_8515_MOESM1_ESM.pdf. [file 12864_2022_8515_MOESM1_ESM.pdf]

(A)

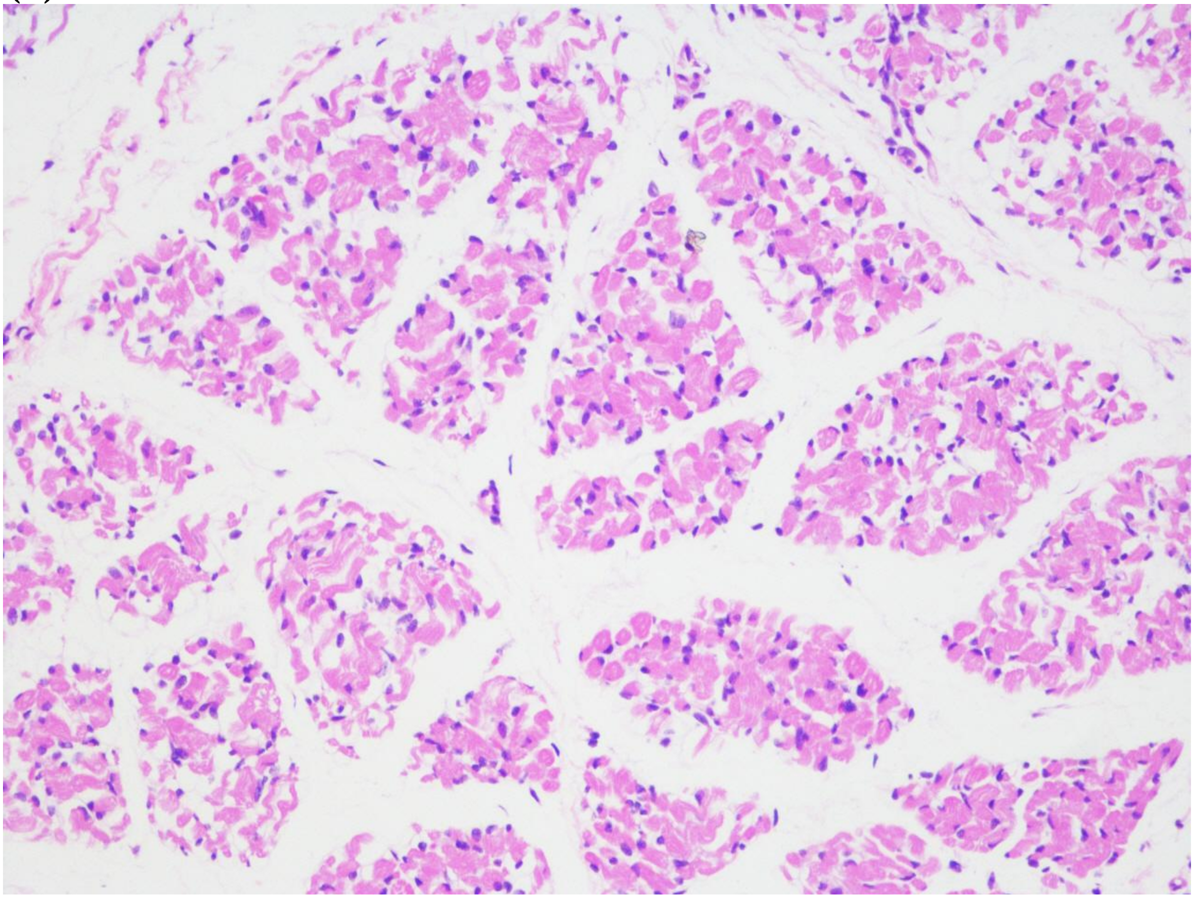

(B)

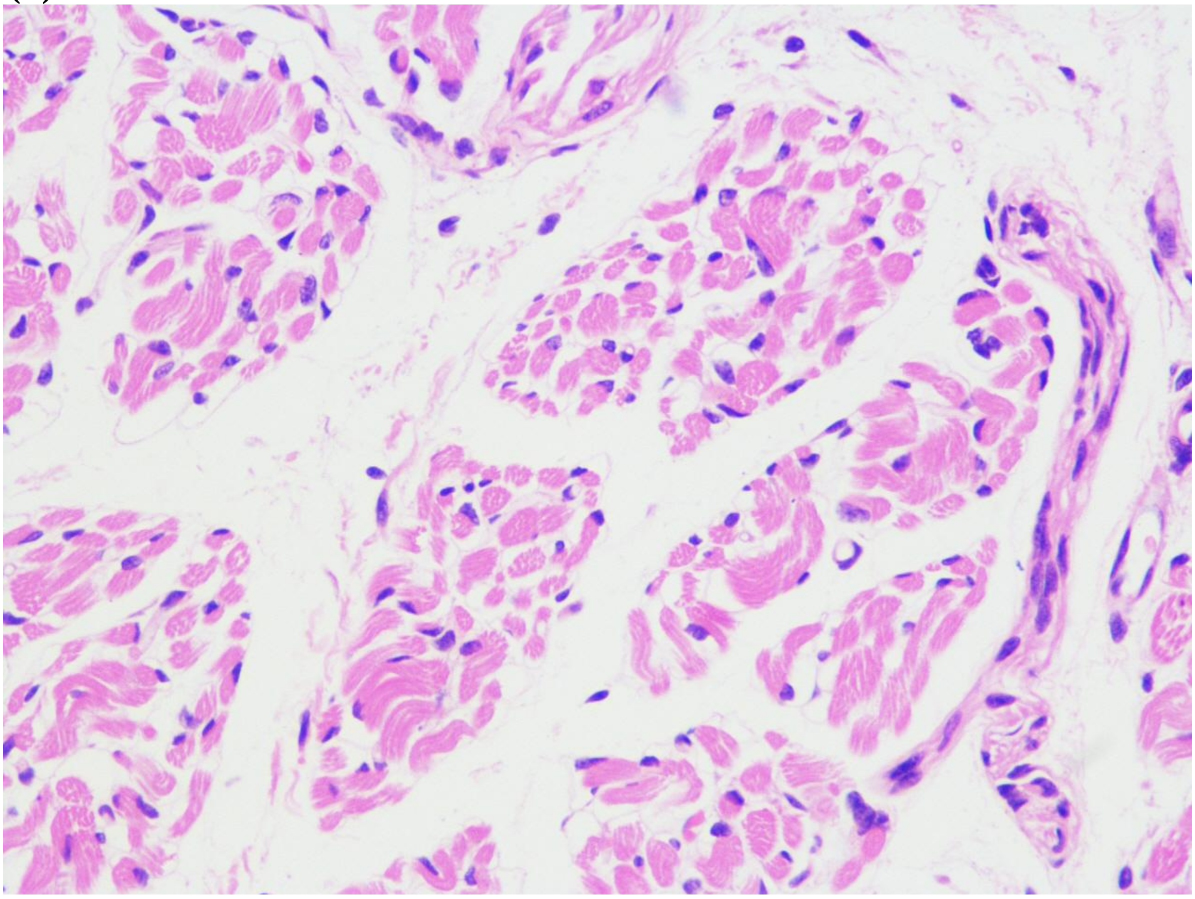

(C)

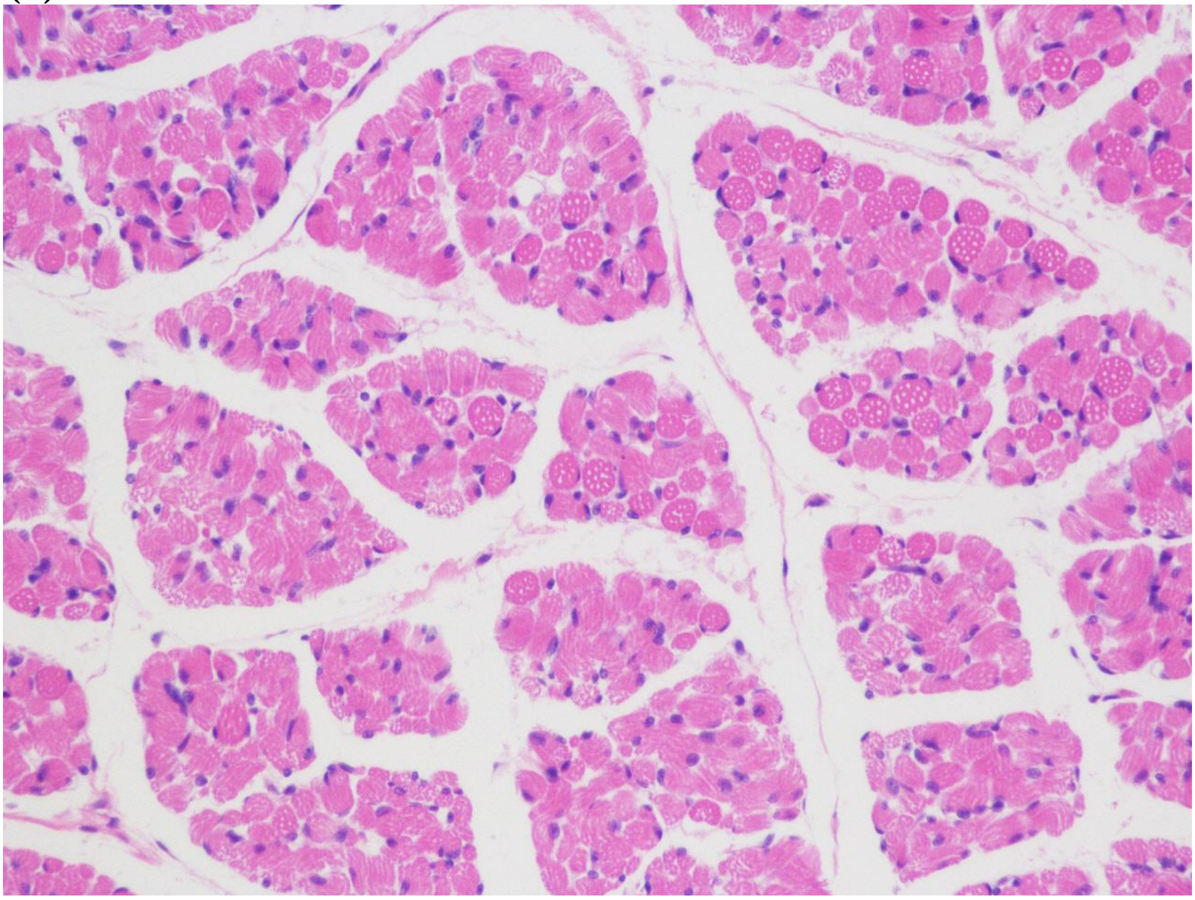

(D)

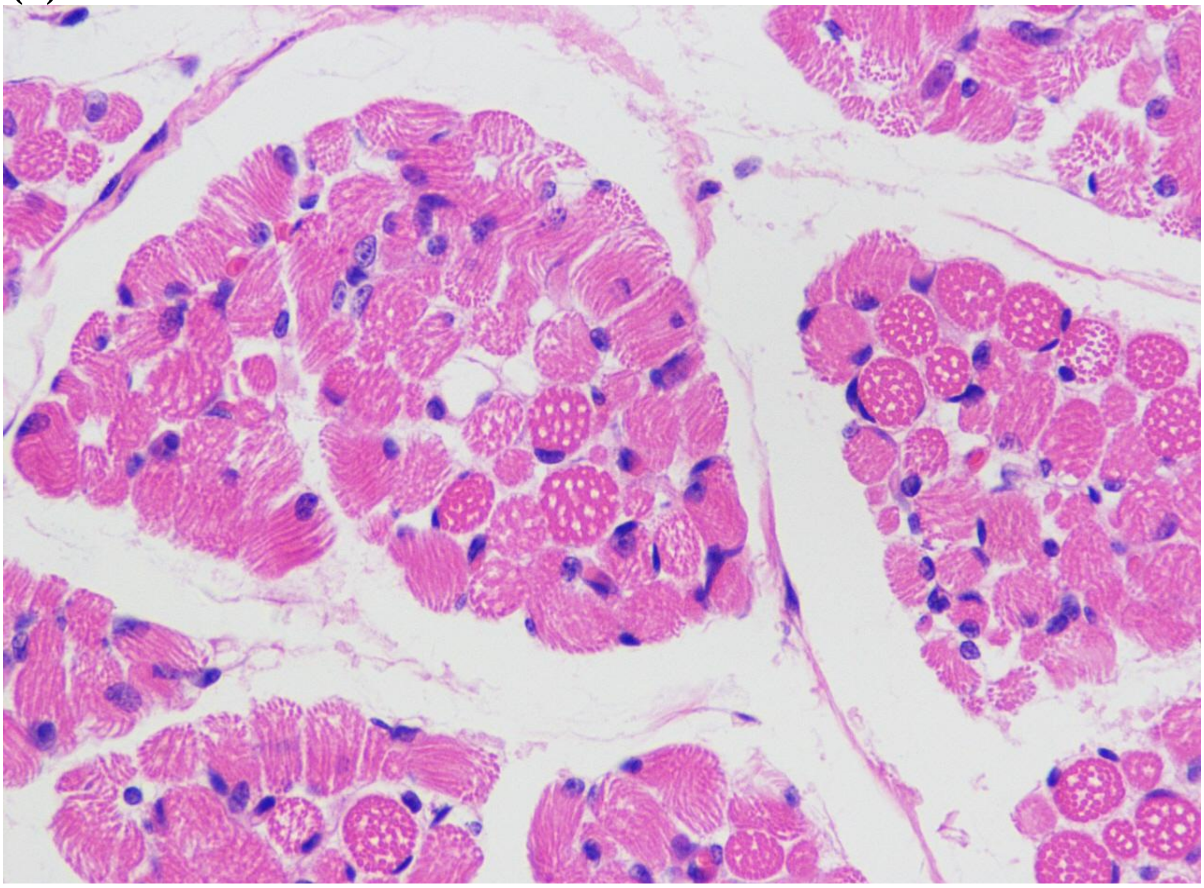

(E)

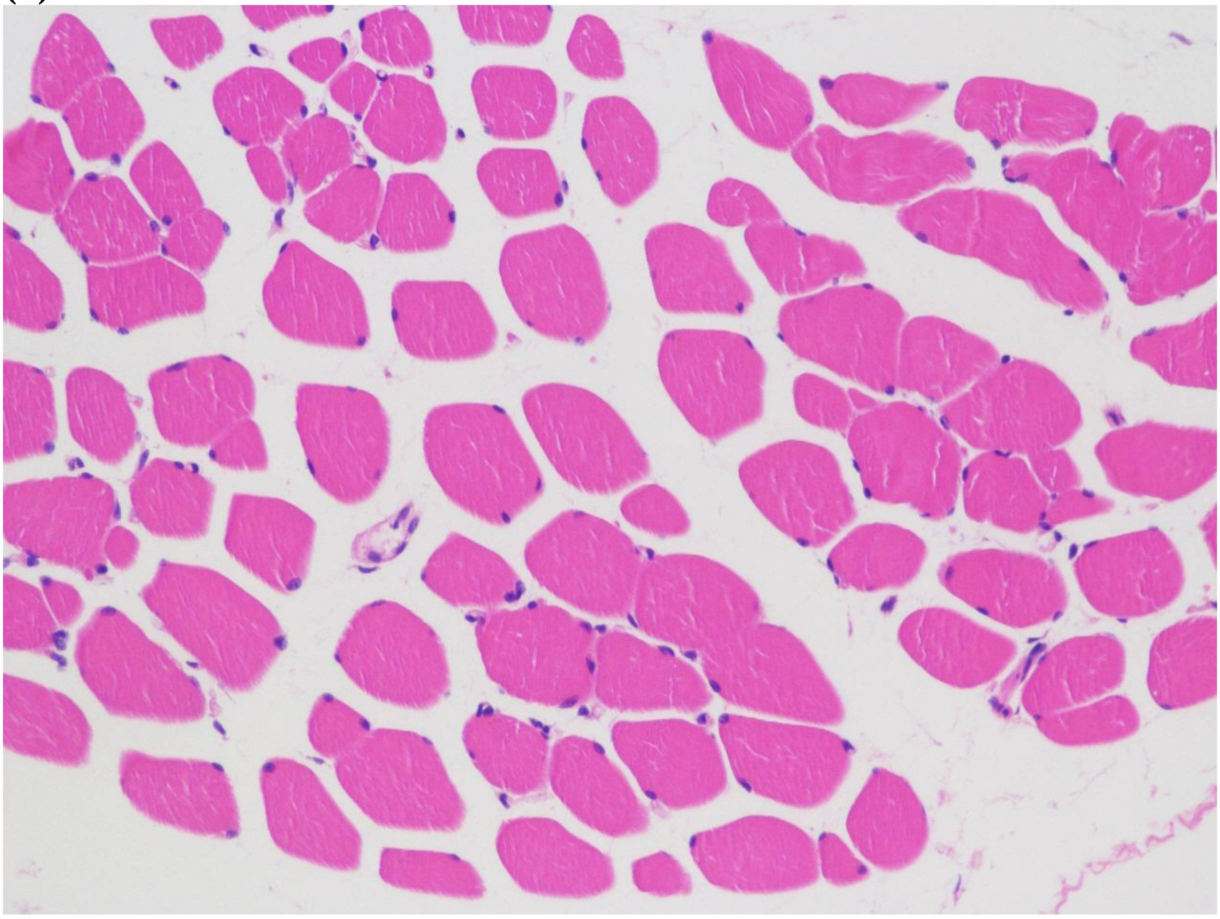

(F)

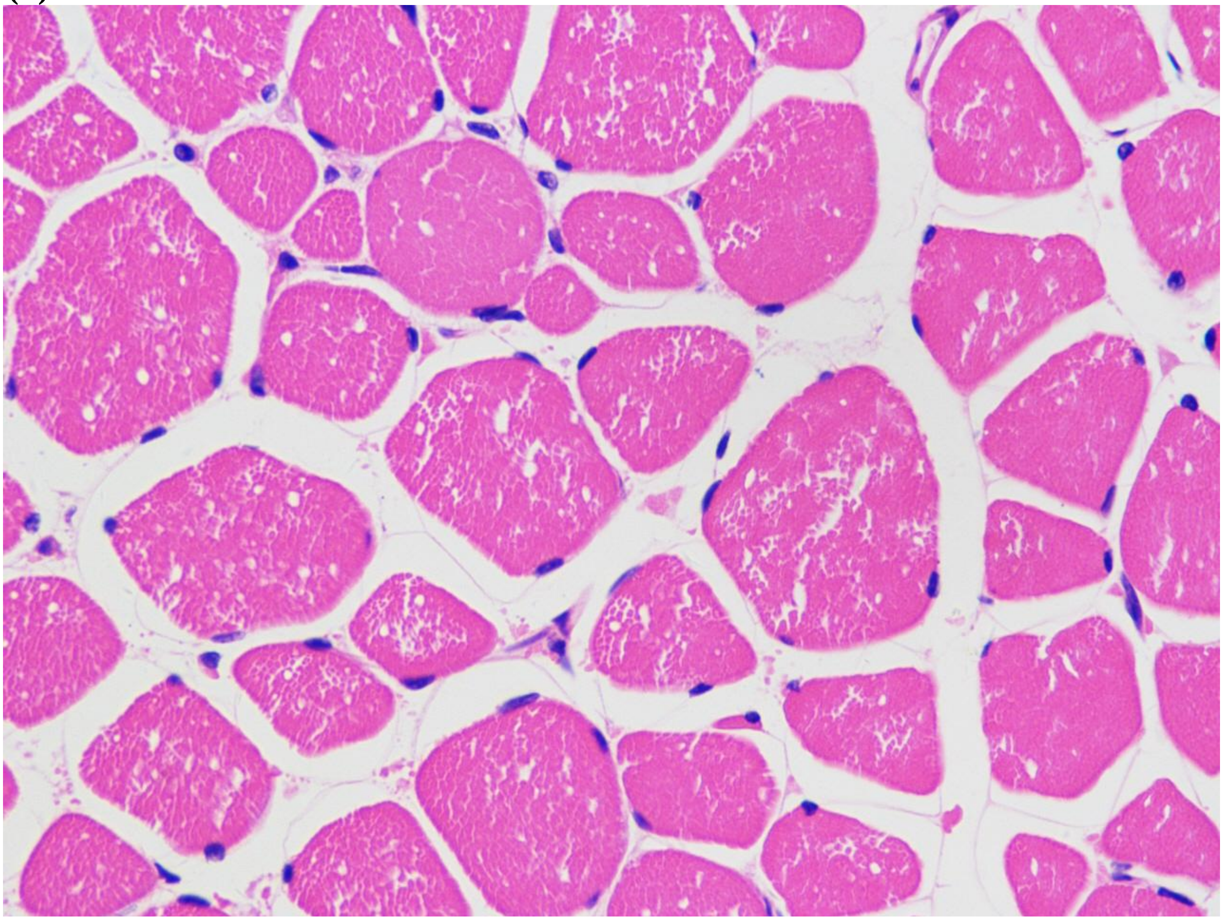

(G)

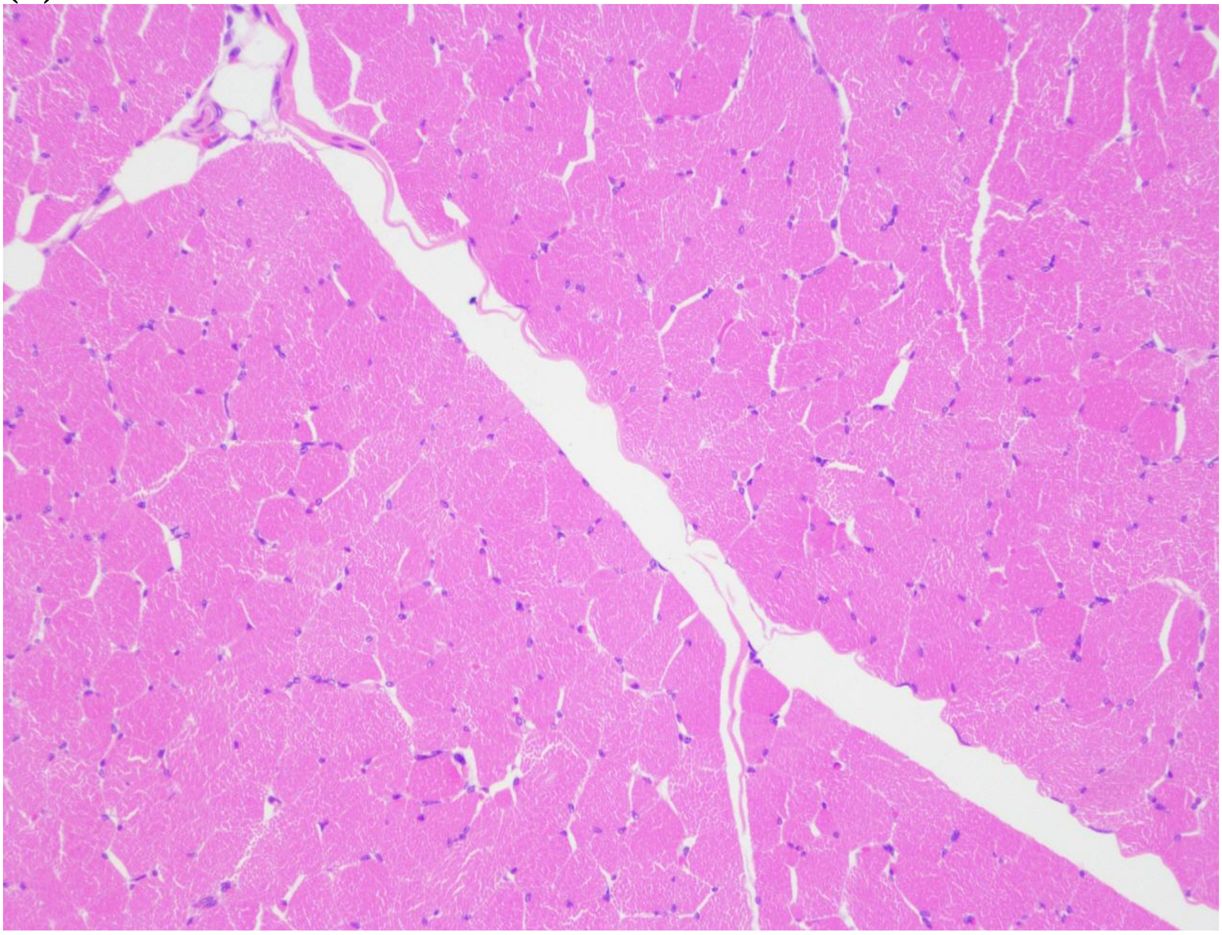

(H)

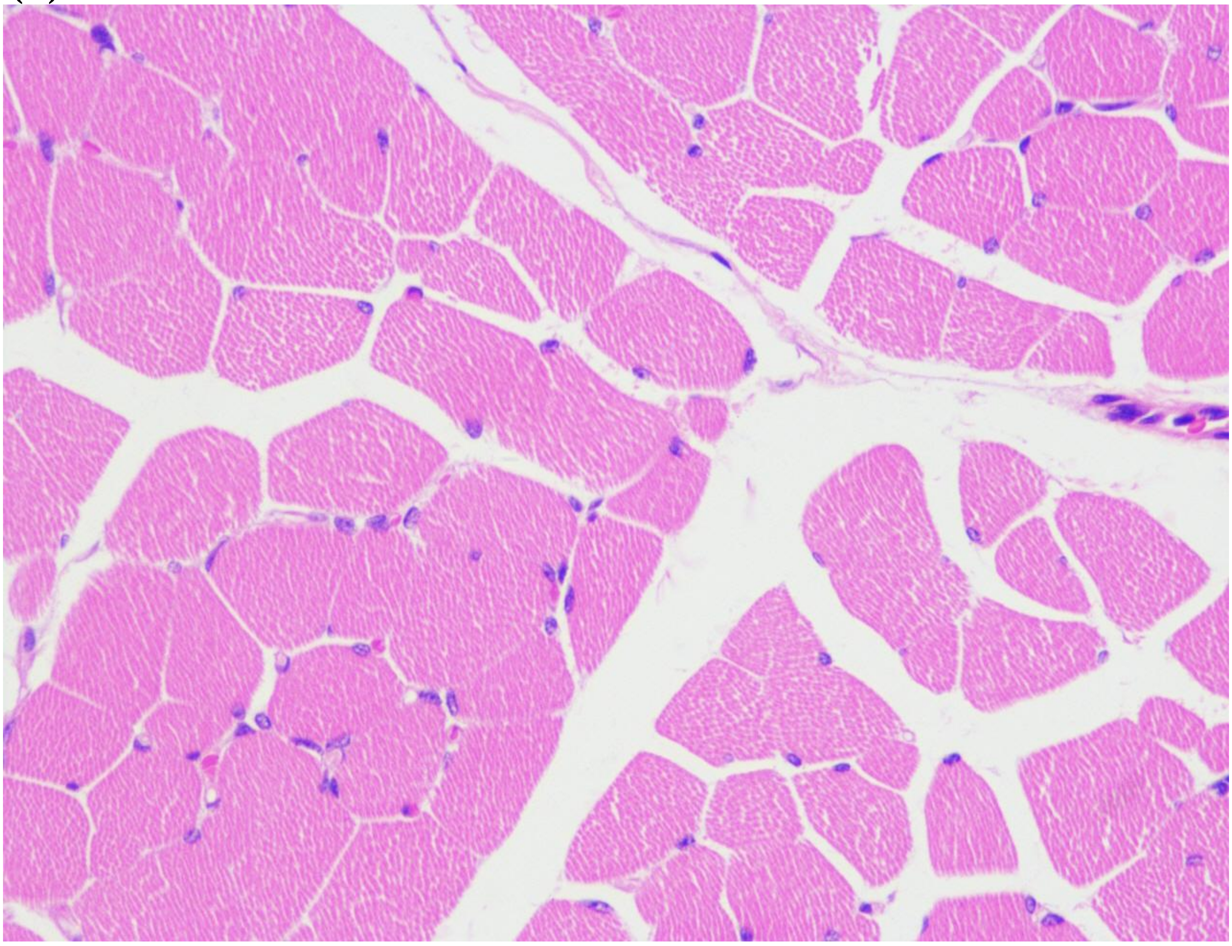

Figure S1A Analysis of the characteristics of muscle fiber between Lantang and Landrace pigs on postnatal day 1 and 90.

Note: The skeletal muscle of Lantang was observed at the first day after birth with hematoxylin-eosin (HE) staining of 200× (A)and 400×(B); The skeletal muscle of Landrace was observed at the first day after birth with HE staining of 200× (C)and 400×(D); The skeletal muscle of Lantang was observed at 90 day after birth with hematoxylin-eosin (HE) staining of 200× (E)and 400×(F); The skeletal muscle of Landrace was observed at 90 day after birth with HE staining of 200× (G)and 400×(H).

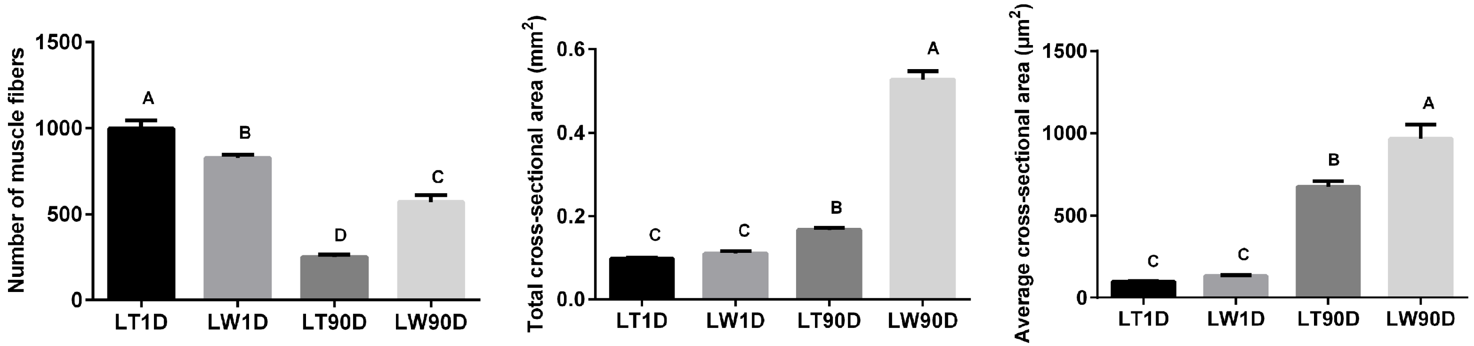

Number of muscle fibers
1500
1000
500
0
A
B
C
D
LT1D
LW1D
LT90D
LW90D
Total cross-sectional area (mm²)
0.6
0.4
0.2
0.0
A
B
C
C
LT1D
LW1D
LT90D
LW90D
Average cross-sectional area (μm²)
1500
1000
500
0
A
B
C
C
LT1D
LW1D
LT90D
LW90D

Figure S1B Number and cross sectional area of myofiber analysed with Image-Pro Plus software in Lantang and Landrace pigs

Note: LT1D, Lantang pig 1 day after birth; LW1D, Landrace pig 1 day after birth; LT90D, Lantang pig 90 day after birth; LW90D, Landrace pig 90 day after birth; different superscripts indicates the significant difference at $P<0.01$.
