# Supplementary material for: Identification of circRNA-associated ceRNA networks using longissimus thoracis of pigs of different breeds and growth stages
Source: BMC Genomics. 2022 Apr 11;23:294. doi: 10.1186/s12864-022-08515-7 (PMC9004053; doi:10.1186/s12864-022-08515-7)
Supplement: Supplementary file 2 — Additional file 2. 12864_2022_8515_MOESM2_ESM.pdf. [file 12864_2022_8515_MOESM2_ESM.pdf]

A

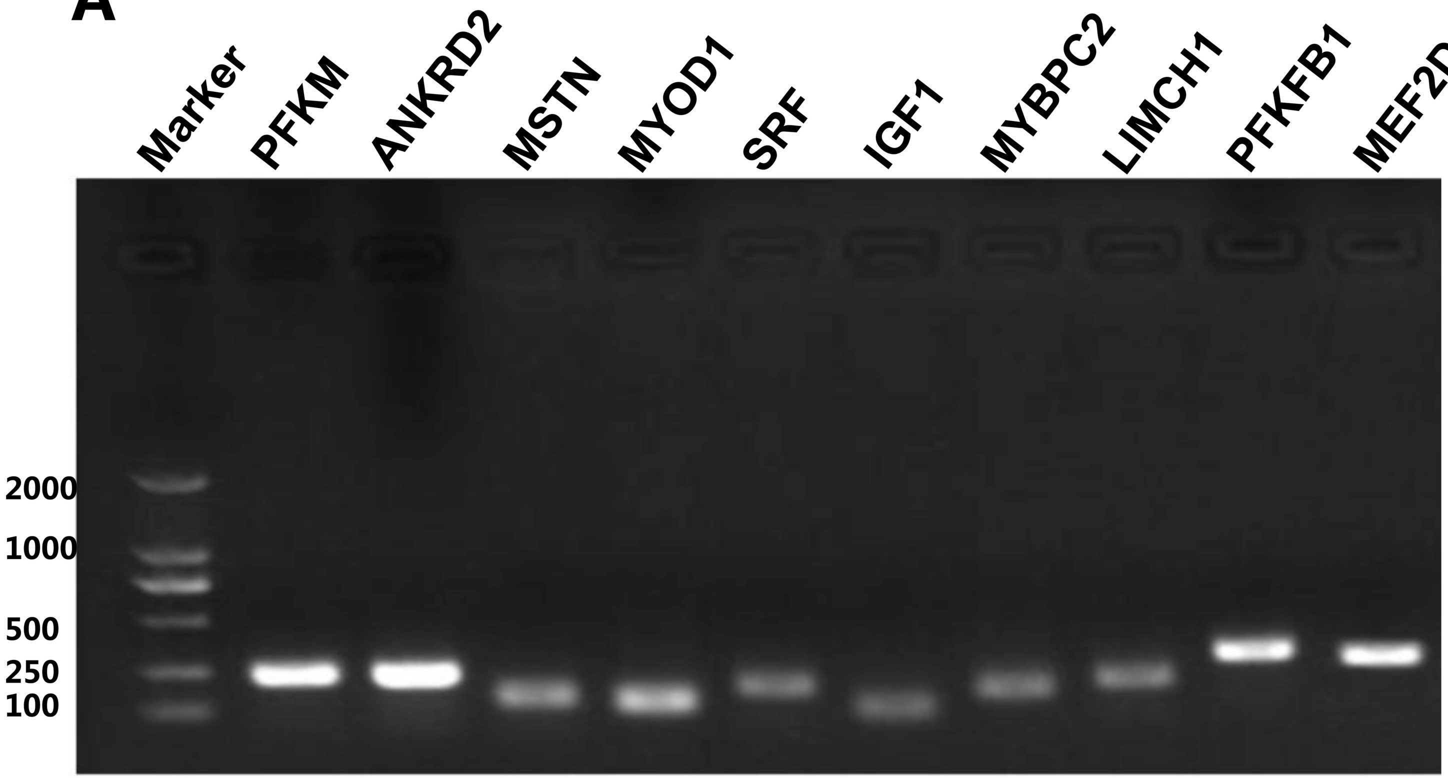
Marker
PFKM
ANKRD2
MSTN
MYOD1
SRF
IGF1
MYBPC2
LIMCH1
PFKFB1
MEF2D
2000
1000
500
250
100

B

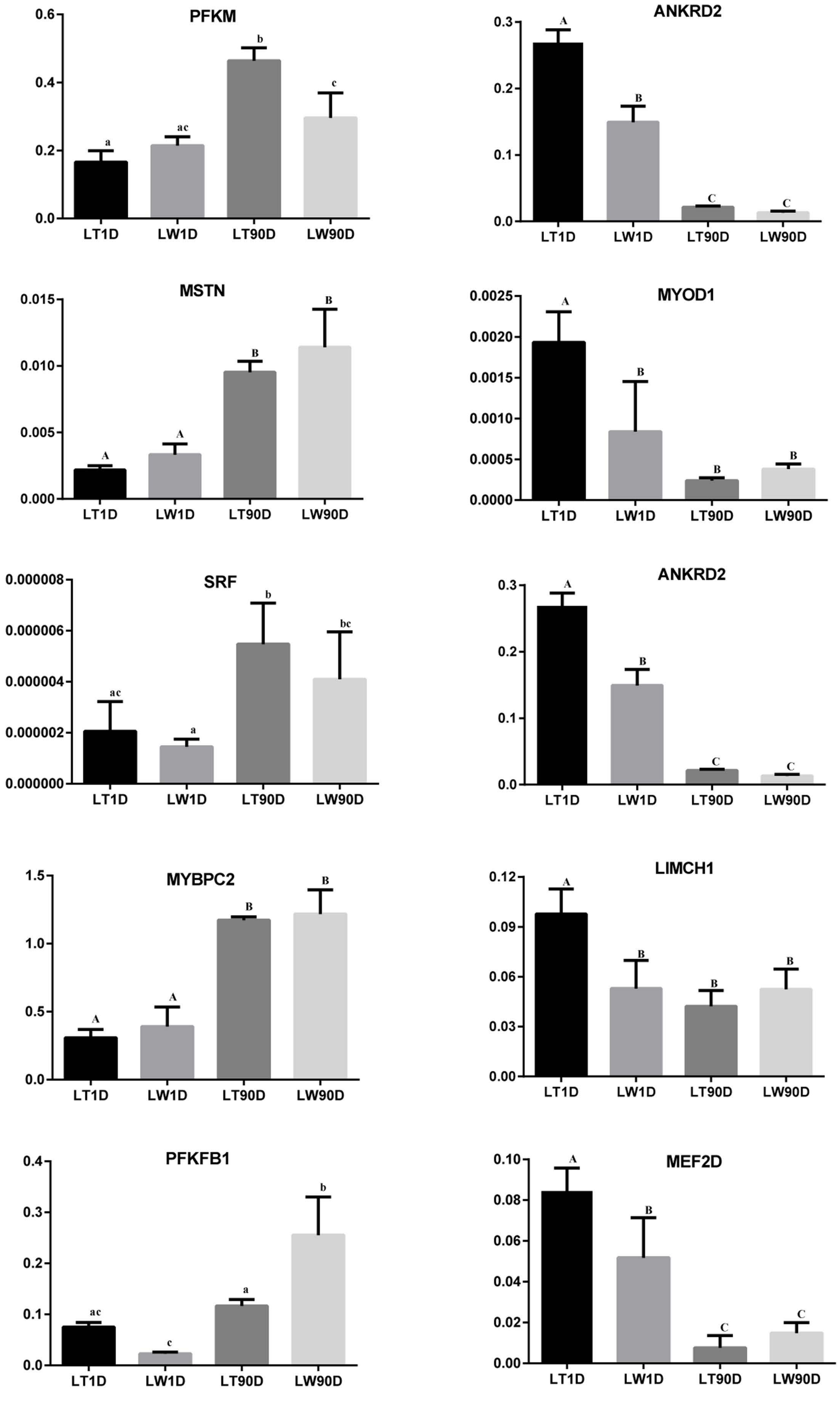
PFKM
0.6
0.4
0.2
0.0
a
ac
b
c
LT1D
LW1D
LT90D
LW90D
ANKRD2
0.3
0.2
0.1
0.0
A
B
C
C
LT1D
LW1D
LT90D
LW90D
MSTN
0.015
0.010
0.005
0.000
A
A
B
B
LT1D
LW1D
LT90D
LW90D
MYOD1
0.0025
0.0020
0.0015
0.0010
0.0005
0.0000
A
B
B
B
LT1D
LW1D
LT90D
LW90D
SRF
0.000008
0.000006
0.000004
0.000002
0.000000
ac
a
b
bc
LT1D
LW1D
LT90D
LW90D
ANKRD2
0.3
0.2
0.1
0.0
A
B
C
C
LT1D
LW1D
LT90D
LW90D
MYBPC2
1.5
1.0
0.5
0.0
A
A
B
B
LT1D
LW1D
LT90D
LW90D
LIMCH1
0.12
0.09
0.06
0.03
0.00
A
B
B
B
LT1D
LW1D
LT90D
LW90D
PFKFB1
0.4
0.3
0.2
0.1
0.0
ac
c
a
b
LT1D
LW1D
LT90D
LW90D
MEF2D
0.10
0.08
0.06
0.04
0.02
0.00
A
B
C
C
LT1D
LW1D
LT90D
LW90D

The expression of 10 coding genes involved in myogenesis using RT-PCR (A) and qPCR (B)

Note: vertical coordinate represented for the relative expression level, and the data represented the mean ± SD from 3 biological replicates with each measurement repeated 3 times. a-c denote values that differ significantly at $P < 0.05$, and A-C denote values that differ significantly at $P < 0.01$.
