# Supplementary material for: Identification of circRNA-associated ceRNA networks using longissimus thoracis of pigs of different breeds and growth stages
Source: BMC Genomics. 2022 Apr 11;23:294. doi: 10.1186/s12864-022-08515-7 (PMC9004053; doi:10.1186/s12864-022-08515-7)
Supplement: Supplementary file 3 — Additional file 3. 12864_2022_8515_MOESM3_ESM.pdf. [file 12864_2022_8515_MOESM3_ESM.pdf]

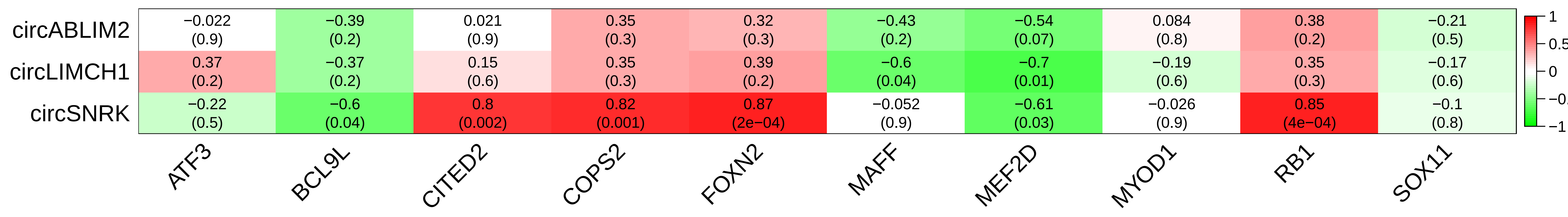
circABLIM2
circLIMCH1
circSNRK
ATF3
BCL9L
CITED2
COPS2
FOXN2
MAFF
MEF2D
MYOD1
RB1
SOX11
−0.022 (0.9)
−0.39 (0.2)
0.021 (0.9)
0.35 (0.3)
0.32 (0.3)
−0.43 (0.2)
−0.54 (0.07)
0.084 (0.8)
0.38 (0.2)
−0.21 (0.5)
0.37 (0.2)
−0.37 (0.2)
0.15 (0.6)
0.35 (0.3)
0.39 (0.2)
−0.6 (0.04)
−0.7 (0.01)
−0.19 (0.6)
0.35 (0.3)
−0.17 (0.6)
−0.22 (0.5)
−0.6 (0.04)
0.8 (0.002)
0.82 (0.001)
0.87 (2e−04)
−0.052 (0.9)
−0.61 (0.03)
−0.026 (0.9)
0.85 (4e−04)
−0.1 (0.8)
1
0
−1

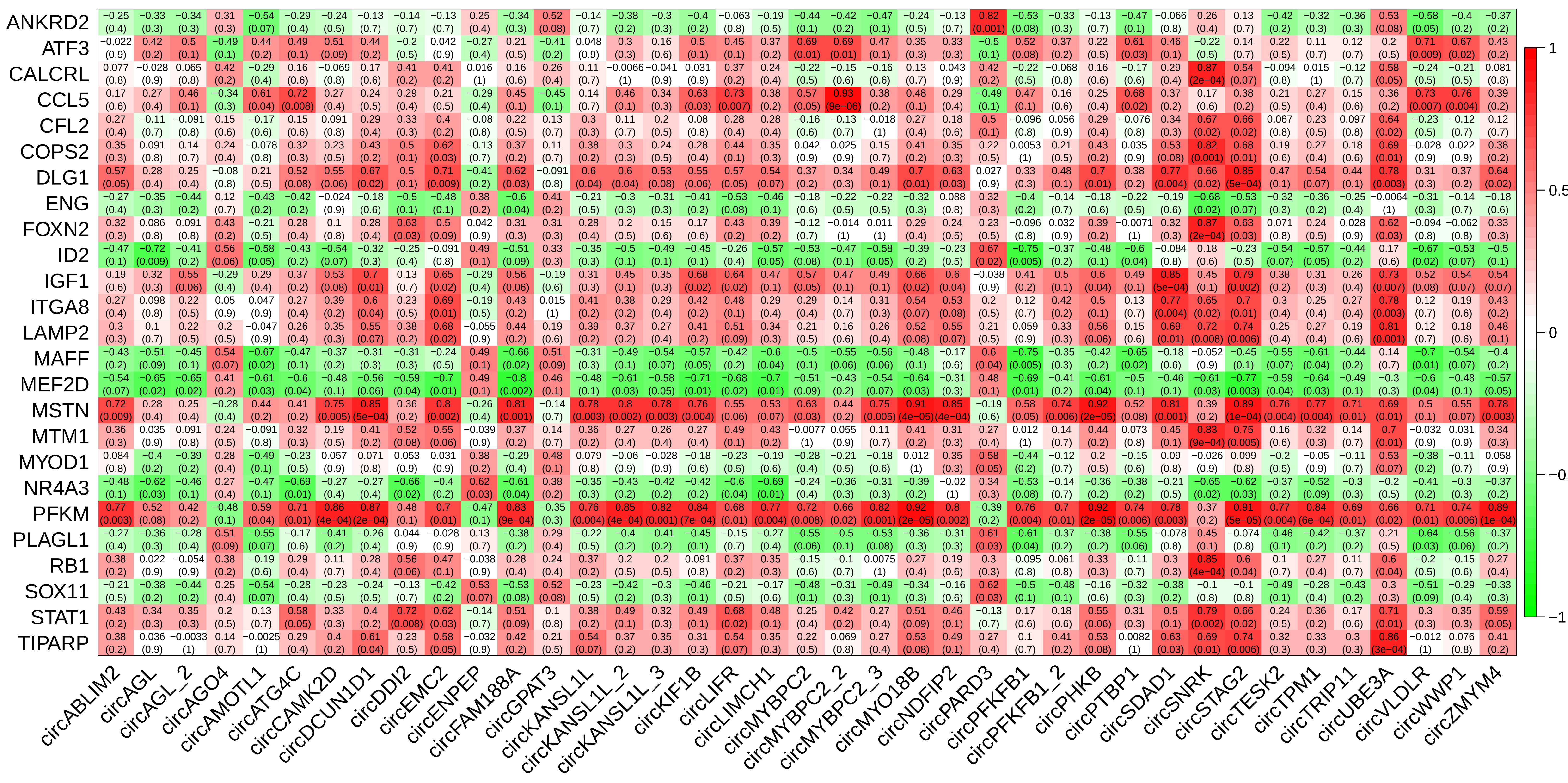
ANKRD2
ATF3
CALCRL
CCL5
CFL2
COPS2
DLG1
ENG
FOXN2
ID2
IGF1
ITGA8
LAMP2
MAFF
MEF2D
MSTN
MTM1
MYOD1
NR4A3
PFKM
PLAGL1
RB1
SOX11
STAT1
TIPARP
circABLIM2
circAGL
circAGL_2
circAGO4
circAMOTL1
circATG4C
circCAMK2D
circDCUN1D1
circDDI2
circEMC2
circENPEP
circFAM188A
circGPAT3
circKANSL1L
circKANSL1L_2
circKANSL1L_3
circKIF1B
circLIFR
circLIMCH1
circMYBPC2
circMYBPC2_2
circMYBPC2_3
circMYO18B
circNDFIP2
circPARD3
circPFKFB1
circPFKFB1_2
circPHKB
circPTBP1
circSDAD1
circSNRK
circSTAG2
circTESK2
circTPM1
circTRIP11
circUBE3A
circVLDLR
circWWP1
circZMYM4
1
0.5
0
−1

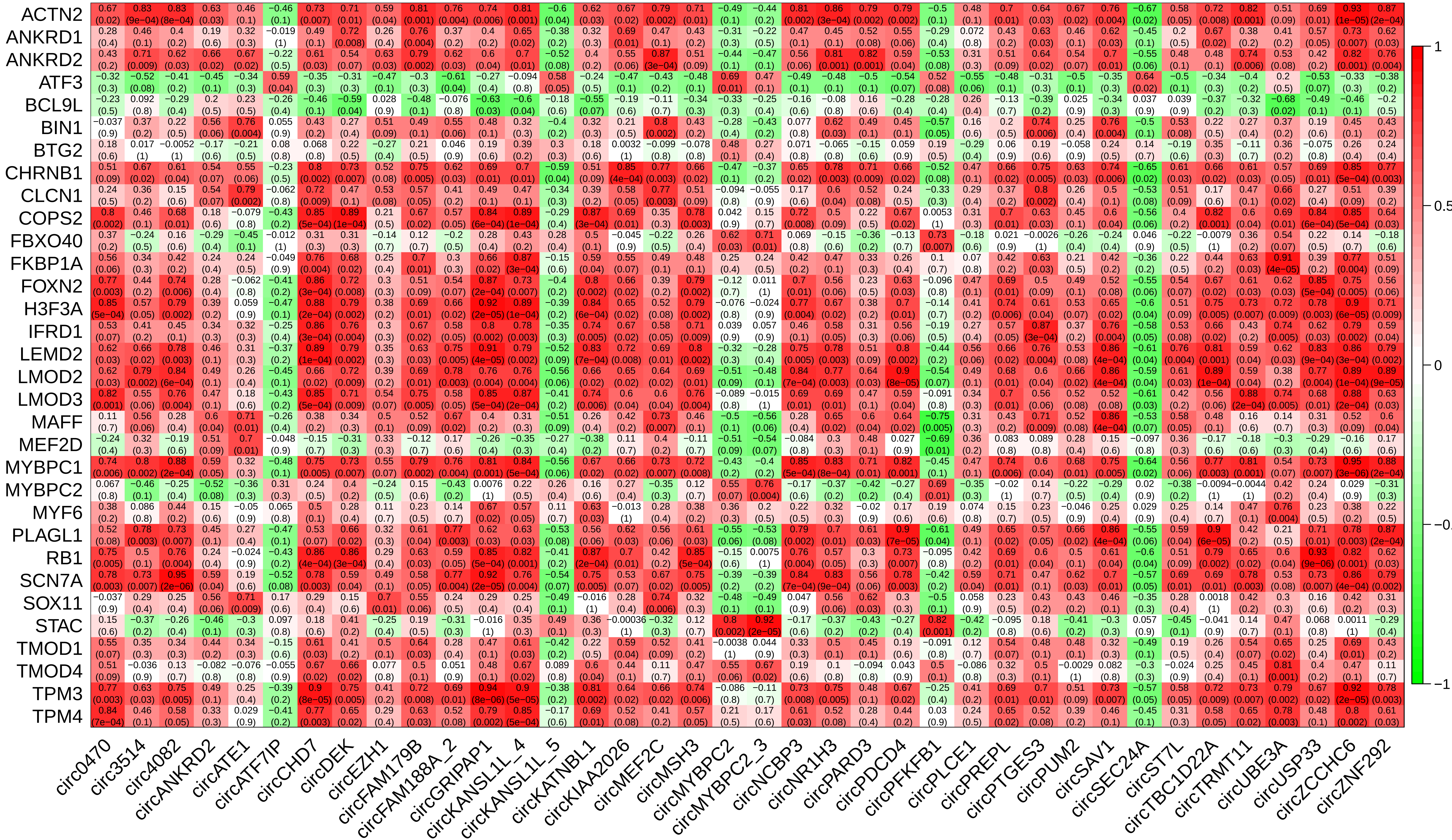
ACTN2
ANKRD1
ANKRD2
ATF3
BCL9L
BIN1
BTG2
CHRNB1
CLCN1
COPS2
FBXO40
FKBP1A
FOXN2
H3F3A
IFRD1
LEMD2
LMOD2
LMOD3
MAFF
MEF2D
MYBPC1
MYBPC2
MYF6
PLAGL1
RB1
SCN7A
SOX11
STAC
TMOD1
TMOD4
TPM3
TPM4
circ0470
circ3514
circ4082
circANKRD2
circATE1
circATF7IP
circCHD7
circDEK
circEZH1
circFAM179B
circFAM188A_2
circGRIPAP1
circKANSL1L_4
circKANSL1L_5
circKATNBL1
circKIAA2026
circMEF2C
circMSH3
circMYBPC2
circMYBPC2_3
circNCBP3
circNR1H3
circPARD3
circPDCD4
circPFKFB1
circPLCE1
circPREPL
circPTGES3
circPUM2
circSAV1
circSEC24A
circST7L
circTBC1D22A
circTRMT11
circUBE3A
circUSP33
circZCCHC6
circZNF292
1
0.5
0
−0.
−1

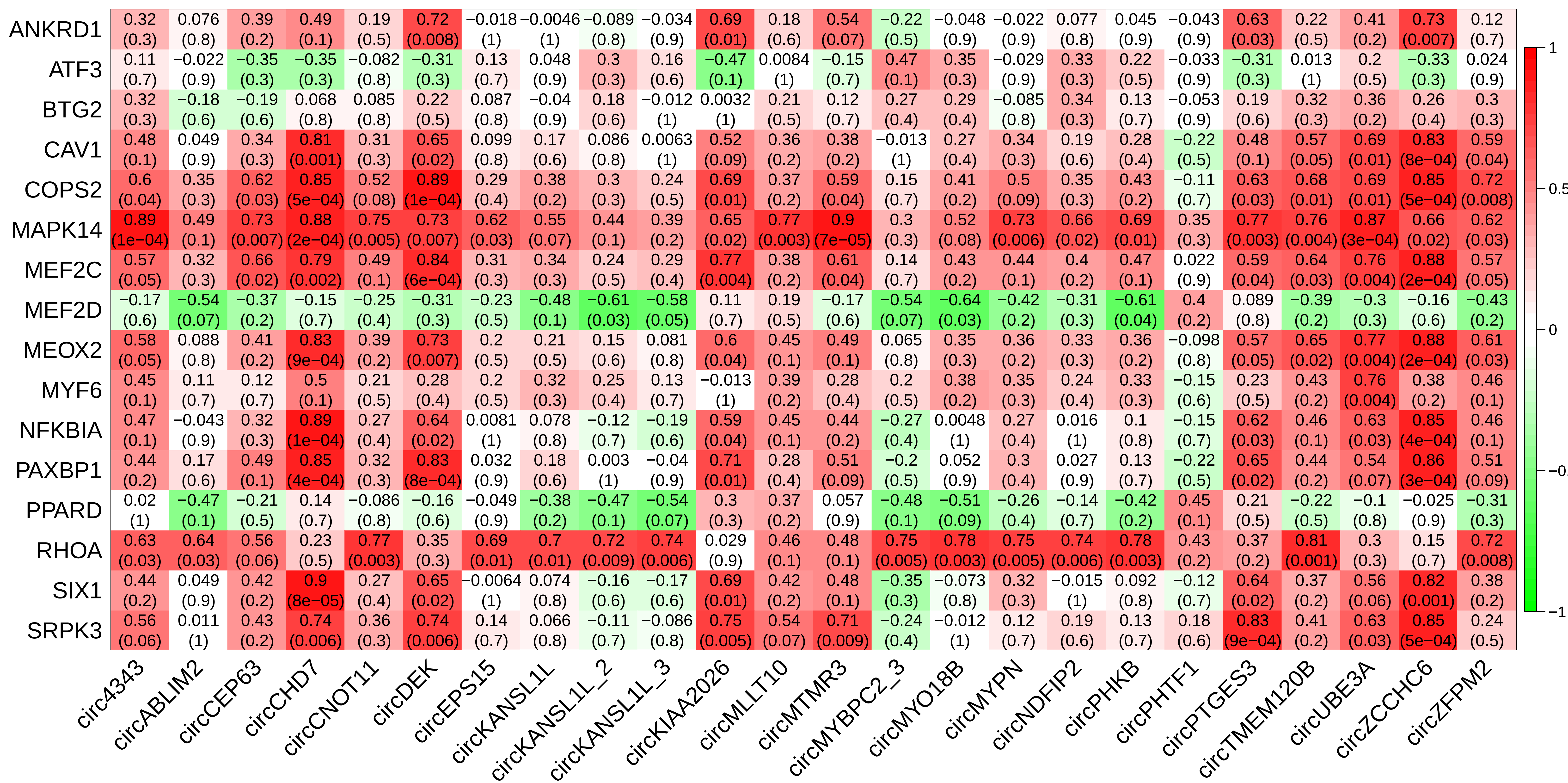
ANKRD1
ATF3
BTG2
CAV1
COPS2
MAPK14
MEF2C
MEF2D
MEOX2
MYF6
NFKBIA
PAXBP1
PPARD
RHOA
SIX1
SRPK3
circ4343
circABLIM2
circCEP63
circCHD7
circCNOT11
circDEK
circEPS15
circKANSL1L
circKANSL1L_2
circKANSL1L_3
circKIAA2026
circMLLT10
circMTMR3
circMYBPC2_3
circMYO18B
circMYPN
circNDFIP2
circPHKB
circPHTF1
circPTGES3
circTMEM120B
circUBE3A
circZCCHC6
circZFPM2
1
0.5
0
−0.[illegible]
−1

Correlation analyses of DE circRNAs with the DE coding genes involved in myogenesis between LT1D and LW1D samples (1st), between LT1D and LT90D samples (2nd), between LW1D and LW90D samples (3rd) and between LT90D and LW90D samples (4th), respectively.

Note: DE: different expressed; Each cell contains the corresponding correlation and *P*-value. The table is colour-coded by correlation according to the colour legend.
