# Supplementary material for: Identification of circRNA-associated ceRNA networks using longissimus thoracis of pigs of different breeds and growth stages
Source: BMC Genomics. 2022 Apr 11;23:294. doi: 10.1186/s12864-022-08515-7 (PMC9004053; doi:10.1186/s12864-022-08515-7)
Supplement: Supplementary file 4 — Additional file 4. 12864_2022_8515_MOESM4_ESM.pdf. [file 12864_2022_8515_MOESM4_ESM.pdf]

A

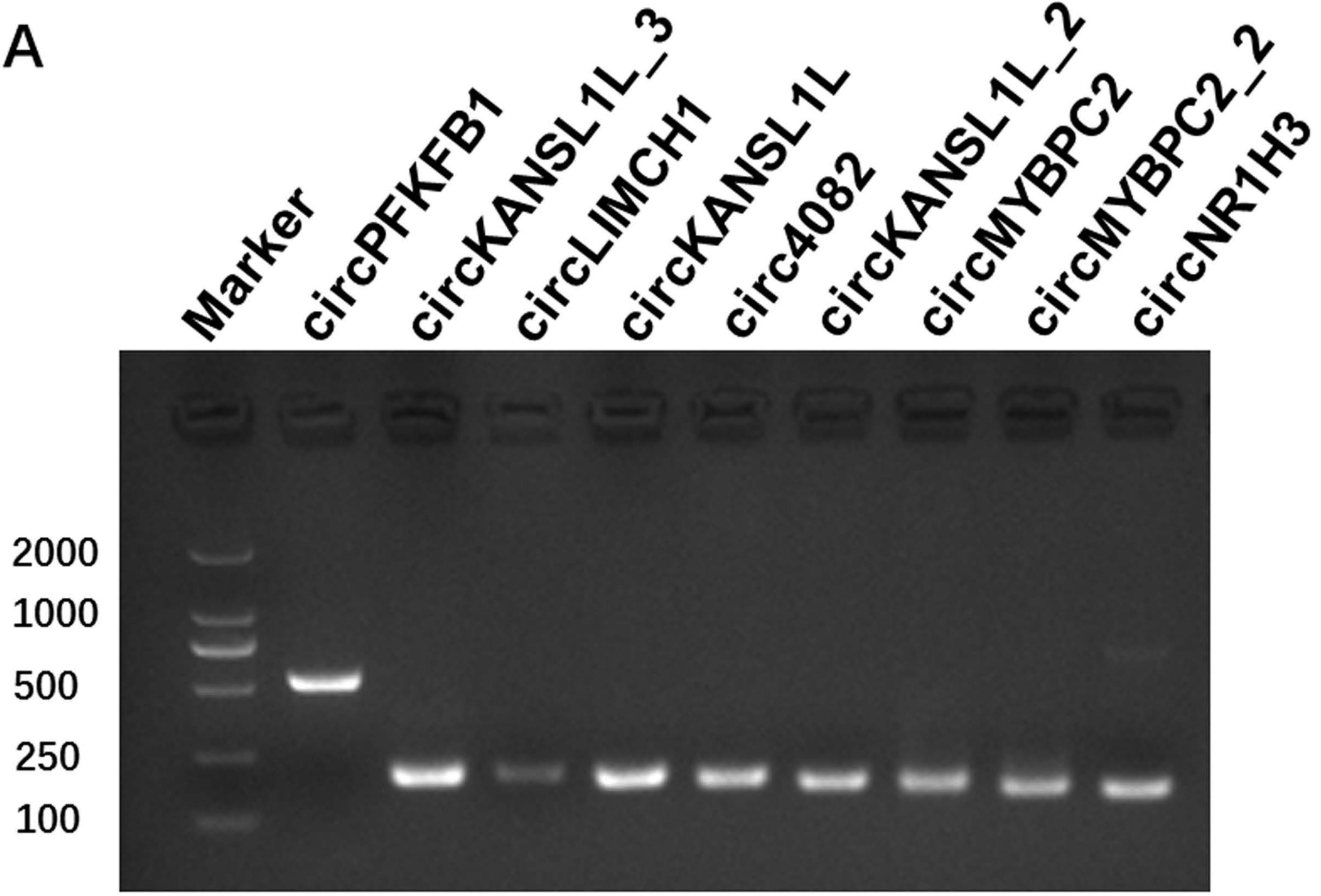

Marker
circPFKFB1
circKANSL1L_3
circLIMCH1
circKANSL1L
circ4082
circKANSL1L_2
circMYBPC2
circMYBPC2_2
circNR1H3
2000
1000
500
250
100

# B

### circPFKFB1 junction sequences (5’ → 3’)

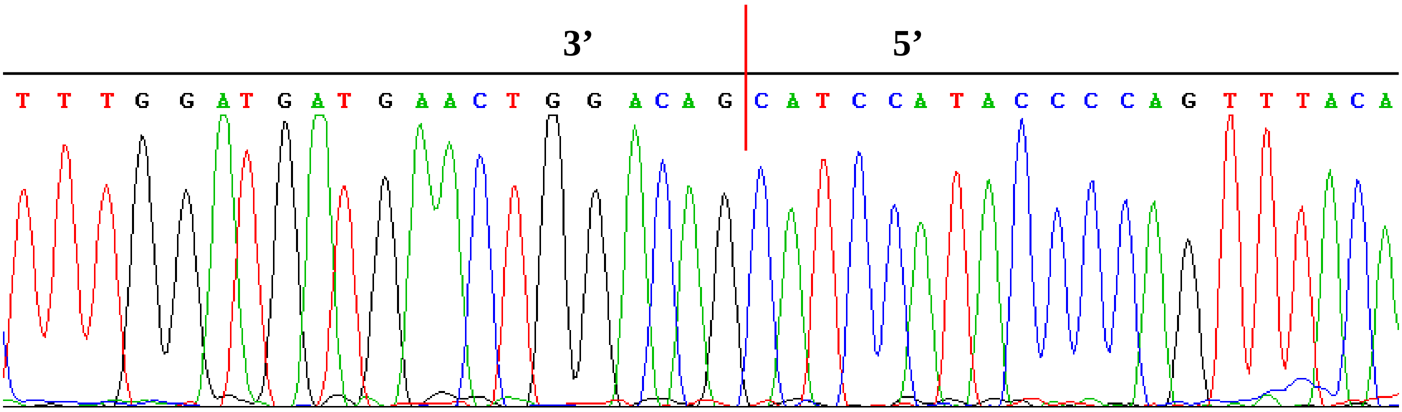


### circKANSL1L junction sequences (5’ → 3’)

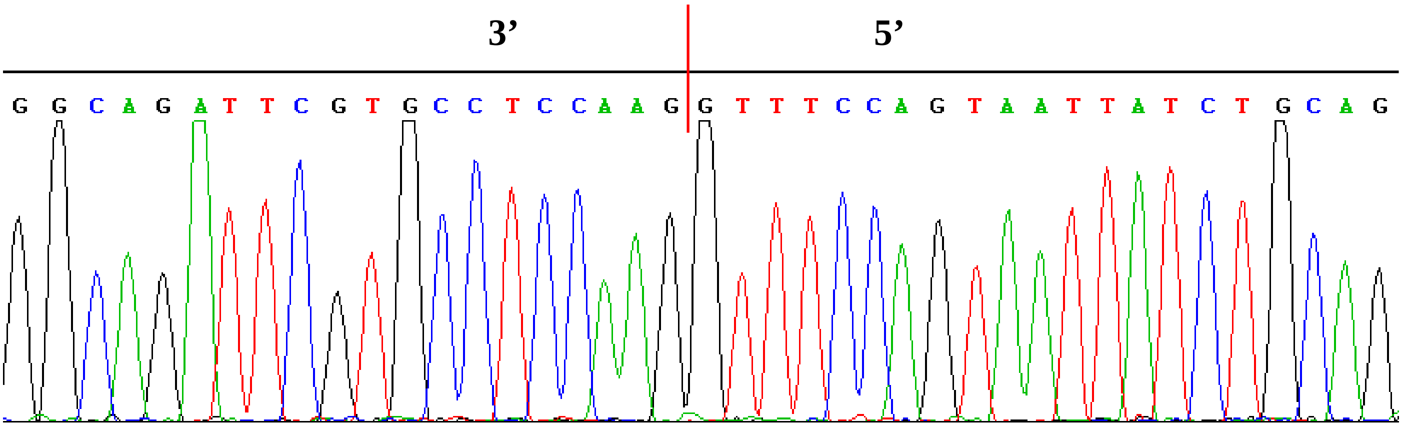


### circLIMCH1 junction sequences (5’ → 3’)

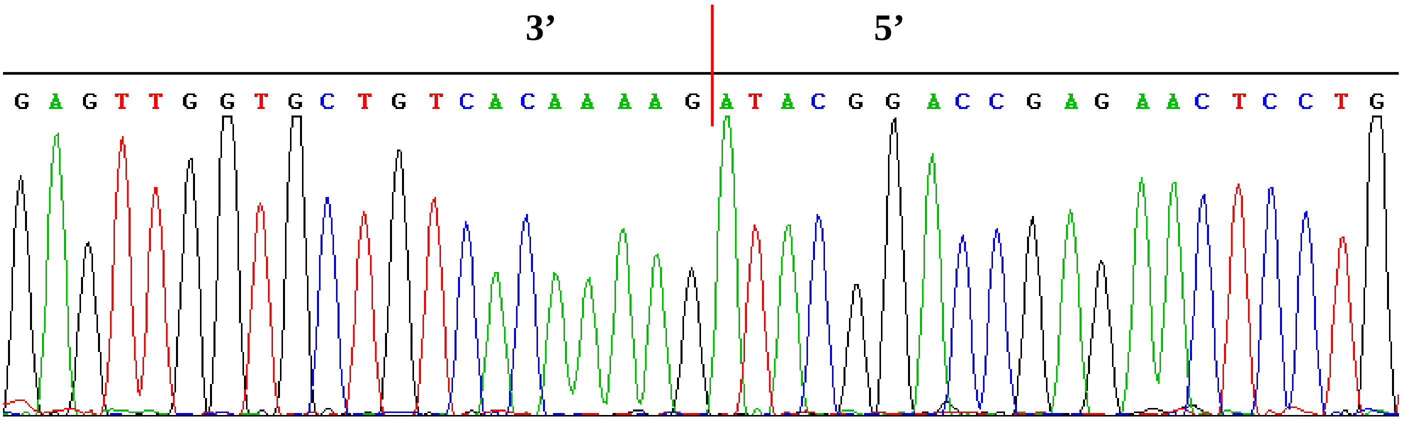

**circKANSL1L_3  junction sequences (5’→3’)**

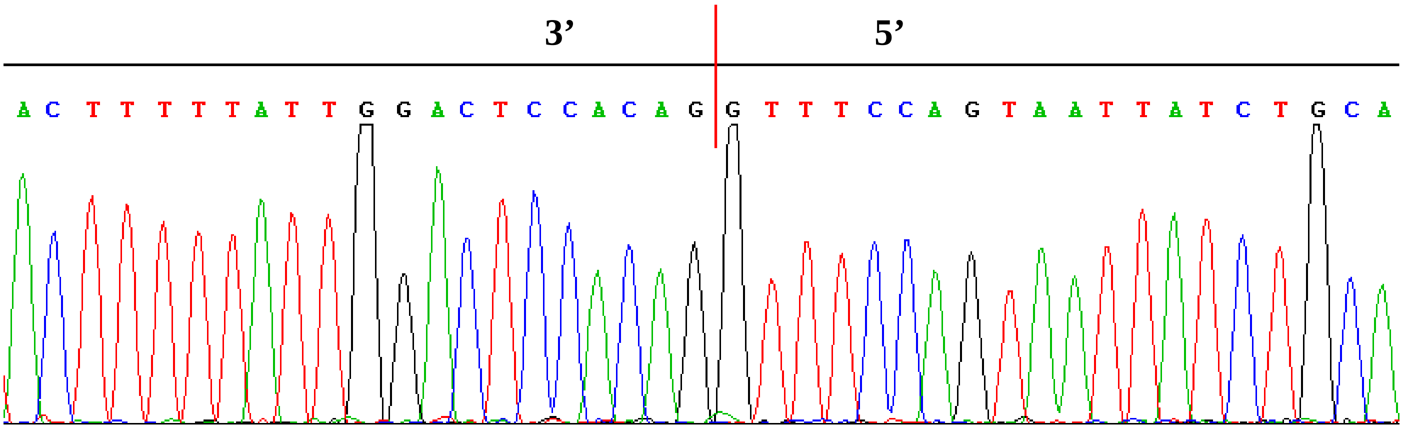


**circ4082  junction sequences (5’→3’)**

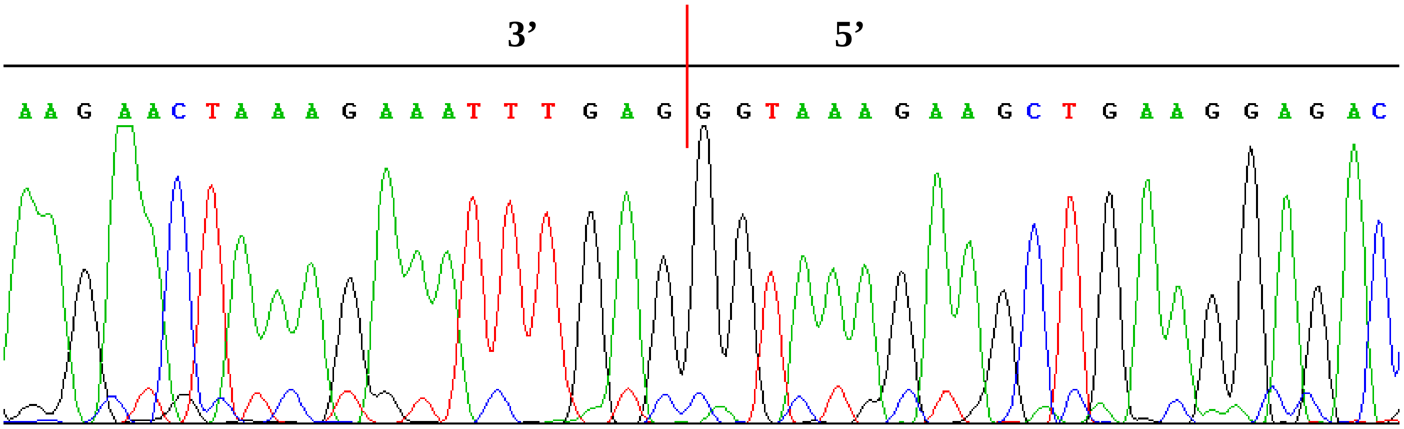


**circKANSL1L_2  junction sequences (5’→3’)**

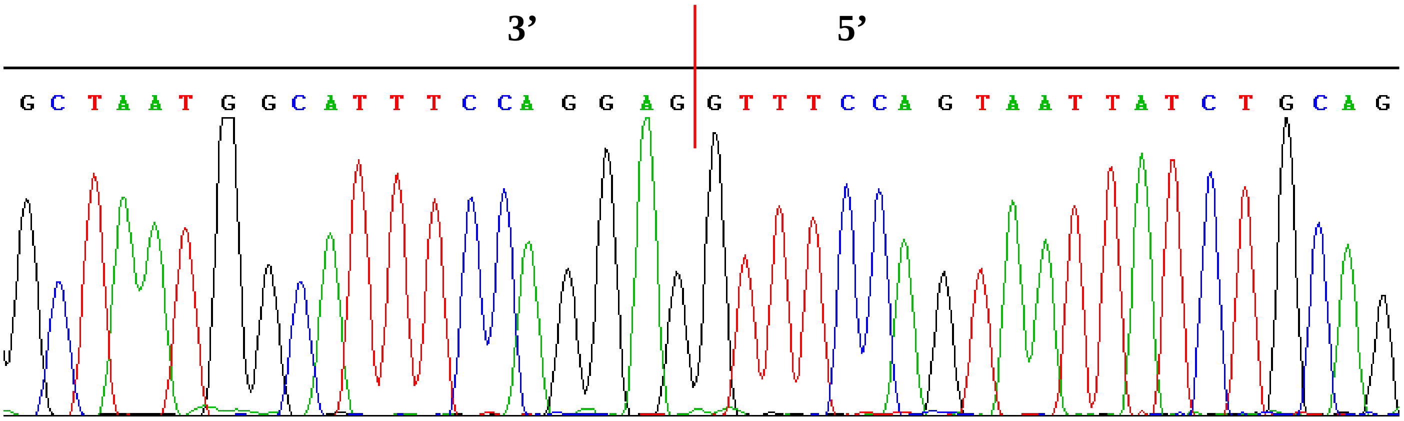

**circMYBPC2 junction sequences (5'→ 3')**

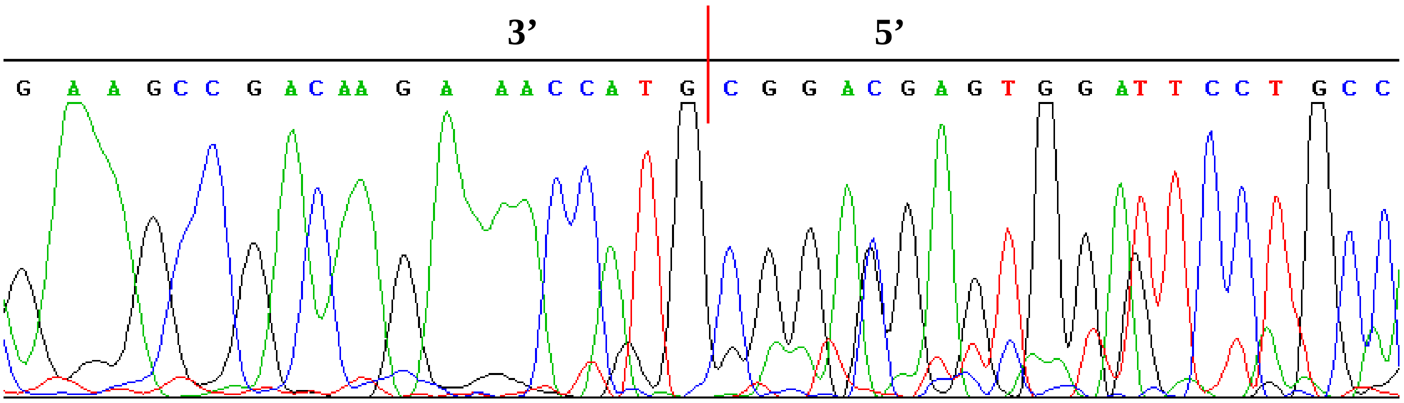


**circMYBPC2_2 junction sequences (5'→ 3')**

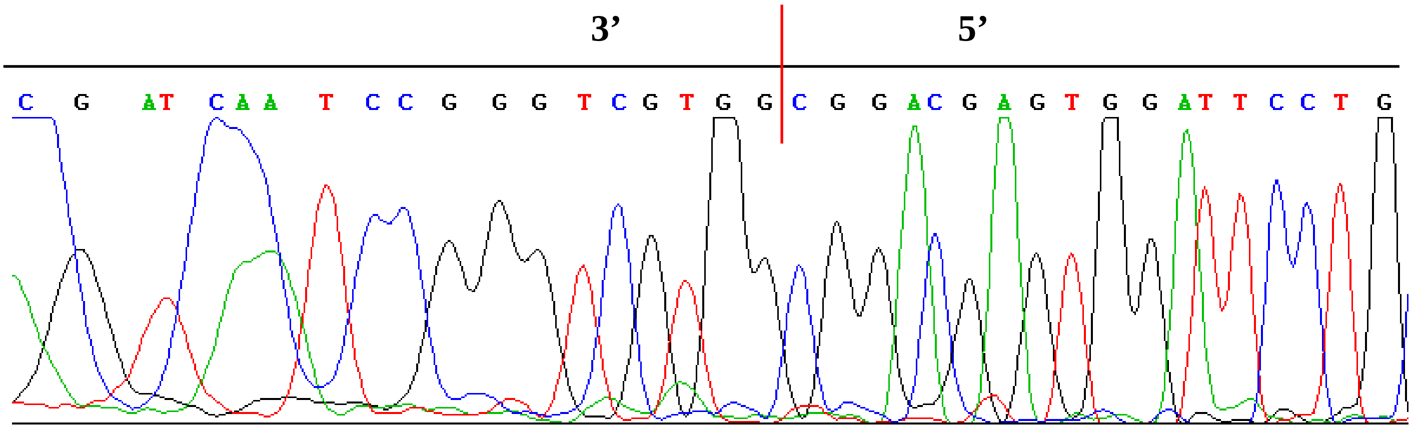


**circNR1H3 junction sequences (5'→ 3')**

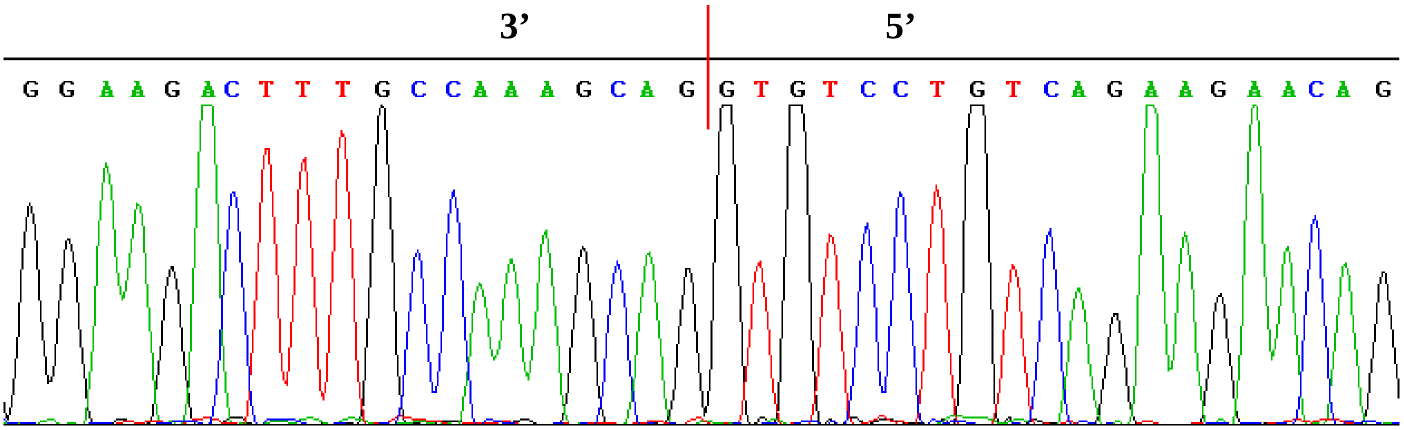


**RT-PCR (A) and Sanger sequencing (B) confirmed head-to-tail junction of circRNA candidates**
