# Supplementary material for: Identification of circRNA-associated ceRNA networks using longissimus thoracis of pigs of different breeds and growth stages
Source: BMC Genomics. 2022 Apr 11;23:294. doi: 10.1186/s12864-022-08515-7 (PMC9004053; doi:10.1186/s12864-022-08515-7)
Supplement: Supplementary file 5 — Additional file 5. 12864_2022_8515_MOESM5_ESM.pdf. [file 12864_2022_8515_MOESM5_ESM.pdf]

Query: pig circKANSL1L form the exon 2 and 3 of ENSSSCT00000025006; Length: 1259

Sbjct: mouse circKANSL1L form the exon 2 and 3 of ENSMUST00000068168; Length: 1262

Score:1524

Identities: 89%

```
Query  1    GTTTCCAGTAATTATCTGCAGGATTCGCCATGACCCCAGCCTTGAGGGAGGCAGCAACAA  60
            ||||||||||||||||||||||||||||||||||||||||  ||| ||||||| ||||||
Sbjct  1    GTTTCCAGTAATTATCTGCAGGATTCGCCATGACCCCAGCTCTGAAGGAGGCAACAACAA  60

Query  61   AGGGTATCTGCTTTTCATCTTTACCAAGTACCATGGAGTCTGACAAGATGCTATGCATGG  120
            |||||||||||||||||||||| |||| ||||||||| |||||||||||||| |||||||
Sbjct  61   AGGGTATCTGCTTTTCATCTTTGCCAAATACCATGGAATCTGACAAGATGCTGTGCATGG  120

Query  121  AAAGTCCAAGAACTGTAGATGAAAAGCT-AA--AAGGAGACACTTTCTCTCAGATGCTGG  177
            ||||||| |||||||||||||||||||| ||   ||||||||||||||||||||||||||
Sbjct  121  AAAGTCCGAGAACTGTAGATGAAAAGCTTAAGGGAGGAGACACTTTCTCTCAGATGCTGG  180

Query  178  GATTTCCAACTCCTGAACCTACTCTTAACACTAATTTTGTGAATTTAAAACATTTTGGCT  237
            ||||||| ||||||||||||||||| || |||||||||||||||||||||||||||| ||
Sbjct  181  GATTTCCGACTCCTGAACCTACTCTAAATACTAATTTTGTGAATTTAAAACATTTTGCCT  240

Query  238  CCCCTCAGTCTTCAAAACATTATCAGACAGTTCTTTTAATGAGTTCTAATGCTACGTTAA  297
            |||||||| |||||||||||| |||||||||||||||||||||||||||| | ||  |||
Sbjct  241  CCCCTCAGGCTTCAAAACATTTTCAGACAGTTCTTTTAATGAGTTCTAATTCAACACTAA  300

Query  298  ATAAATACAATGAGCATTATAAACAAAAGAAATTAGGGGAACCCAACTGCAATAAGCTGA  357
            |||| || |||||| ||||||| ||||||||| ||  |||  ||||||||| ||| ||||
Sbjct  301  ATAAGTATAATGAGAATTATAACCAAAAGAAAGTAATGGAGTCCAACTGCAGTAAACTGA  360

Query  358  AAAACTTACTGTGTAATGGCAGCAATGTTCAGCTCAGTAAAATCTGTCATTCTCATTCTG  417
            ||||  ||||||| ||||||||||   ||||||||||||| ||||| | |||||| || |
Sbjct  361  AAAATGTACTGTGCAATGGCAGCAGCATTCAGCTCAGTAAGATCTGCCCTTCTCACTCCG  420

Query  418  AA---GAGTTCATCAAAAAGGAACCTCTGTCAGATACCACAAGTCAGTGCATGACAGATG  474
            ||   ||||||||||||||||||| ||  || ||||||||||| |||||||||| |||| 
Sbjct  421  AAAACGAGTTCATCAAAAAGGAAC-TC--TCGGATACCACAAGCCAGTGCATGAAAGATA  477

Query  475  TACAAATTATTTTGGATTCAAATATAACCAAAGACACTAATGTAGATAAAGTACAACTGC  534
            |||||||  || ||||||||||| | ||||||||| |||||||||| | | |||| ||||
Sbjct  478  TACAAATCGTTCTGGATTCAAATCTCACCAAAGACGCTAATGTAGACAGACTACATCTGC  537
```

```
Query  535   AAAACTATAAGTGGTACCAAAAGAATGCACTTTTAGATAAAGTTAATGATGCTGAGATTA  594
             |||||| ||| ||||||||||||||||||||||| |||||| | | |||| || ||||||
Sbjct  538   AAAACTGTAAATGGTACCAAAAGAATGCACTTTTGGATAAATTCACTGATACTAAGATTA  597

Query  595   AAAAGGCTTTATTGCACTGTGCTCAAAACAAAATTGGGCCTGGCCAGTCAAATGTGCCTA  654
             |||||| ||||||||| ||| ||||||| |||||||| ||| |||| ||| |||||||||
Sbjct  598   AAAAGGGTTTATTGCAATGTACTCAAAAGAAAATTGGACCTAGCCACTCAGATGTGCCTA  657

Query  655   TTAGTTCCTCAGCTGCTGAAAAAGAGGAGGAAGTGAATGCTCGTTTACTTCATTGTGTAA  714
              ||||||||||||||||||||||||| ||||||||||||||||||||||||||||||| |
Sbjct  658   CTAGTTCCTCAGCTGCTGAAAAAGAGCAGGAAGTGAATGCTCGTTTACTTCATTGTGTGA  717

Query  715   GCAAACAGAAAATTTTACTTAGCCAGGCTAAAAGAACTCAGAAACATTTGCAGATGCTCC  774
             | |||||||||||||||||||||||||||| ||||||||||||||| |||||||||||||
Sbjct  718   GTAAACAGAAAATTTTACTTAGCCAGGCTAGAAGAACTCAGAAACACTTGCAGATGCTCC  777

Query  775   TGGCAAAGCATGTTGTTAAGCACTATGGTCAGCAAATGAAATTTTCTATGAAACATCAAC  834
             ||||||||||||||||||||||||||||||| || ||||||||||||||||||||||| |
Sbjct  778   TGGCAAAGCATGTTGTTAAGCACTATGGTCAACAGATGAAATTTTCTATGAAACATCAGC  837

Query  835   TCCCCAGAACGAAGAGTTTTCACGAACATACCACAGTTTCGGA-TAACAGTTTACCTAAA  893
             | |||| || |||||  ||||| ||||  || |||||| | || ||| ||||||| | ||
Sbjct  838   TGCCCACAATGAAGATCTTTCATGAACCCACAACAGTT-CTGAGTAATAGTTTACTTGAA  896

Query  894   TGCACTGAAATTAAACCAGACATCAATATATTGACTACAGAGAATAAATTGTGGACTGAT  953
               |||||||||||| |||||  |||| |||||| || |||||||||| || |||  ||||
Sbjct  897   CACACTGAAATTAAGCCAGAAGTCAACATATTGGCTTCAGAGAATAAGTTTTGGGATGAT  956

Query  954   ACCAAAAATGGCTTTGCACGGTGTACAGCTGCAGAAATCCAAAGATTTGCACTGTCTGCT  1013
             || || ||||||||| | | ||||||||||||||||||||||||||||||||||||||||
Sbjct  957   ACAAACAATGGCTTTTCTCAGTGTACAGCTGCAGAAATCCAAAGATTTGCACTGTCTGCT  1016

Query  1014  TCAGGGCTGTTGTCTCATGTTGAAGAGGGCCTGGATTCTGATGCAACTGATAGCAGTTCT  1073
              |||||||||||||||||||||||||||| ||||| ||||| || || |||||||| ||
Sbjct  1017  ACAGGGCTGTTGTCTCATGTTGAAGAGGGTCTGGACTCTGACGCCACCGATAGCAGCTCA  1076

Query  1074  GATGACGATTTGGATGAATATACCATTAGAAAAAATGTGGCAGTTAACTGTAGTACTGAA  1133
             |||||||| ||||||||||||||||||||||||||||||||||| |||| ||||||||||
Sbjct  1077  GATGACGAGTTGGATGAATATACCATTAGAAAAAATGTGGCAGTCAACTCTAGTACTGAA  1136

Query  1134  TGGAAGTGGCTTGTAGACAGAGCAAGAGTTGGCAGCCGATGGACATGGCTTCAAGCCCAG  1193
             ||||||||||||||||||||||||  |||||||||||||||||| ||||||||||| |||
Sbjct  1137  TGGAAGTGGCTTGTAGACAGAGCACAAGTTGGCAGCCGATGGACTTGGCTTCAAGCTCAG  1196
```

```
Query  1194  ATTTCAGAACTAGAGTATAAAATCCAACAACTAACAGATGTTCACAGGCAGATTCGTGCC  1253
             |||||||| || || || |||||||| || ||||||||| | ||||||||||||||||||
Sbjct  1197  ATTTCAGAGCTGGAATACAAAATCCAGCAGCTAACAGATATCCACAGGCAGATTCGTGCC  1256

Query  1254  TCCAAG  1259
             ||||||
Sbjct  1257  TCCAAG  1262
```

The similarity comparison of circKANSL1L sequences between pig and mouse
